# Why halides enhance heterogeneous metal ion charge transfer reactions†

**DOI:** 10.1039/d1sc03642d

**Published:** 2021-08-26

**Authors:** Jacob Florian, Harsh Agarwal, Nirala Singh, Bryan R. Goldsmith

**Affiliations:** Department of Chemical Engineering, University of Michigan Ann Arbor Michigan 48109-2136 USA bgoldsm@umich.edu snirala@umich.edu; Catalysis Science and Technology Institute, University of Michigan Ann Arbor Michigan 48109-2136 USA

## Abstract

The reaction kinetics of many metal redox couples on electrode surfaces are enhanced in the presence of halides (*i.e.*, Cl^−^, Br^−^, I^−^). Using first-principles metadynamics simulations, we show a correlation between calculated desorption barriers of V^3+^–anion complexes bound to graphite *via* an inner-sphere anion bridge and experimental V^2+^/V^3+^ kinetic measurements on edge plane pyrolytic graphite in H_2_SO_4_, HCl, and HI. We extend this analysis to V^2+^/V^3+^, Cr^2+^/Cr^3+^, and Cd^0^/Cd^2+^ reactions on a mercury electrode and demonstrate that reported kinetics in acidic electrolytes for these redox couples also correlate with the predicted desorption barriers of metal–anion complexes. Therefore, the desorption barrier of the metal–anion surface intermediate is a descriptor of kinetics for many metal redox couple/electrode combinations in the presence of halides. Knowledge of the metal–anion surface intermediates can guide the design of electrolytes and electrocatalysts with faster kinetics for redox reactions of relevance to energy and environmental applications.

## Abstract 2

Halide-induced rate enhancements are correlated with the desorption barriers of aqueous metal–anion complexes on electrodes, which can guide electrode and electrolyte selection to enhance redox kinetics of metal ion charge transfer reactions.

## Graphical abstract

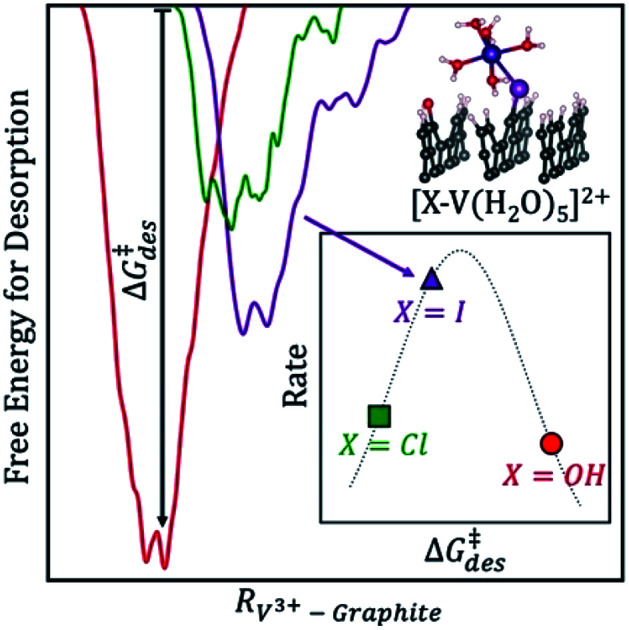

## Introduction

**Fig. 1:**
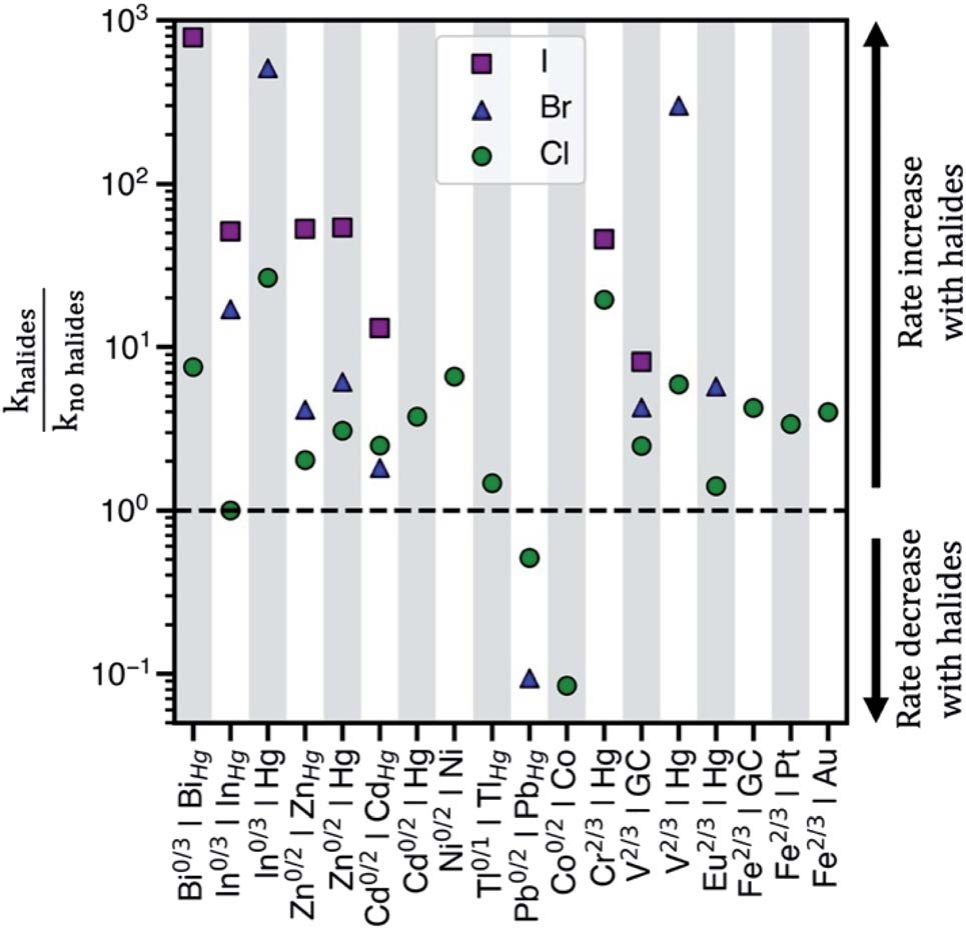
The observed ratios of rate constants with and without halides for various redox couples and electrodes in aqueous electrolytes. *k*_halides_ corresponds to the rate constant in the presence of halides, whereas *k*_no halides_ corresponds to the rate constant in sulfate or perchlorate electrolytes. The abscissa shows ‘redox couple|electrode surface’ combinations where rate data has been reported. The halides present in the electrolytes considered are either chloride, bromide, or iodide. GC = glassy carbon electrode and *M*_Hg_ = metal–Hg amalgam alloy electrode. Table S1 in the ESI† contains the standard rate constants for these redox couples.

Electrochemical charge transfer of metal ions has applications in energy storage,^1^ wastewater remediation,^2^ organic synthesis,^3^ and chemical production.^4^ Understanding and controlling charge transfer at the electrode surface would increase energy efficiency and product selectivity, and reduce capital cost of devices. Interestingly, halides accelerate the kinetics of many electrochemical reactions in aqueous solution, Fig. 1. For example, heterogeneous charge transfer reactions including V^2+^/V^3+^,^5,6^ Cr^2+^/Cr^3+^,^7,8^ Fe^2+^/Fe^3+^,^9,10^ and Eu^2+^/Eu^3+^,^11^ and metal electrodeposition reactions, such as Cd^0^/Cd^2+^ and Zn^0^/Zn^2+^,^12,13^ show rate constants (*k*) that are up to 10^3^ higher in the presence of chloride (Cl^−^), bromide (Br^−^), or iodide (I^−^). Kinetic enhancement by halides is observed on many electrodes, including glassy carbon (GC),^14^ Hg,^15^ Au,^7,10^ and Pt,^9,16^ where the increase in *k* typically is the largest for I^−^, followed by Br^−^, and then Cl^−^. Thus, understanding the cause of these enhancements would guide electrolyte and electrode selection.

Halide-induced rate enhancements may arise from the halide ions being adsorbed on electrodes to serve as sites for adsorption and charge transfer of metal ions. This mechanism, whereby the halide anion adsorbed on the electrode acts as a bridge in the electron transfer between metal ion and electrode, has been called anion bridging.^15^ The anion bridging mechanism has been invoked to explain rate enhancements for Cr^2+^/Cr^3+^, V^2+^/V^3+^, Sb^3+^/Sb^5+^, and Fe^2+^/Fe^3+^ redox couples.^10,14,15,17,18^ A two-step inner-sphere mechanism for how an adsorbed anion (*X) promotes oxidation of a metal ion (M^*n*+^) through the formation of an adsorbed metal–anion intermediate (*XM^*n*+1^) is written in eqn (1) and (2). Although here we write M^*n*+1^ as the product leaving behind *X, it is also possible that the halide becomes part of the desorbed complex as XM^*n*+1^.1
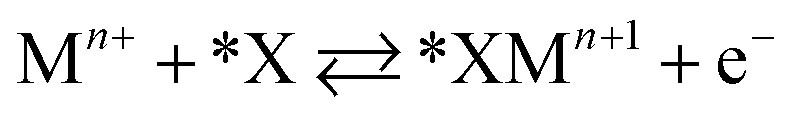
2
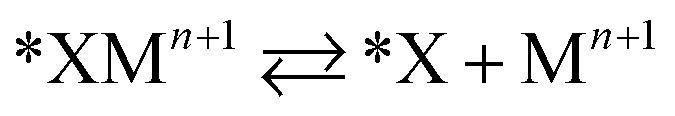


Although this mechanism is plausible, there is little knowledge of how the metal ion and the bridging anion together promote charge transfer and why the rate enhancement relative to non-complexing electrolytes (*i.e.*, through *OH bridge) typically follows the order of Cl^−^ < Br^−^ < I^−^. This order of rate enhancement correlates with increasing free anion polarizability,^19,20^ that is, polarizable anions can more easily transfer one of their outer shell electrons to the metal cation while the other electron is being transferred from the electrode surface to the anion bridge. However, free anion polarizability does not account for the electrode and redox couple identity. Interfacial potential shifts due to anion adsorption can also increase the rate of metal ion redox couples, as has been reported previously.^21^ However, some studies have found that electrostatic effects alone are insufficient to explain the large increases (*k*_halide_/*k*_no halides_ > 10) in the observed rate constants and postulated that anion bridging may be responsible.^8,10,15,18^

**Scheme 1:**
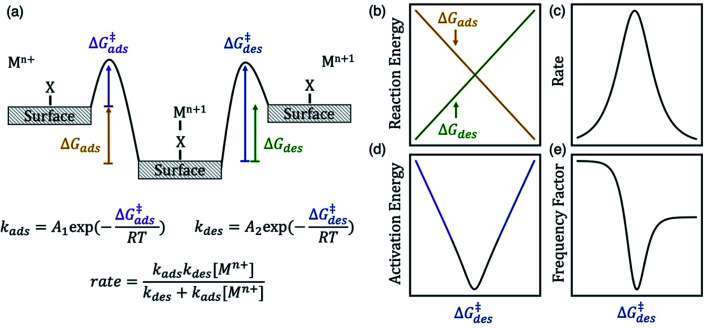
(a) Energy diagram of a metal ion charge transfer reaction involving an adsorbed intermediate. The desorption free energy barrier (Δ*G*^‡^_des_) of the metal–anion surface intermediate can be a descriptor for kinetics of a M^*n*+^/M^*n*+1^ redox couple. The diagrams on the right show the dependence of (b) reaction energy, (c) rate (proportional to exchange current density and observed rate constant), (d) apparent activation energy, and (e) apparent frequency factor on Δ*G*^‡^_des_, assuming Brønsted–Evans–Polanyi scaling relations hold. The rate law derivation is included in the ESI.† Here *k*_ads_ has units of s^−1^, *k*_des_ has units of mol L^−1^ s^−1^, and overall rate has units of mol L^−1^ s^−1^.

Herein, we test our hypothesis that anion bridging on electrodes increases the kinetics for many metal redox couples by changing the energy of the surface intermediate (*XM^*n*+1^) and its adsorption and desorption barriers. The energy of *XM^*n*+1^ controls the fraction of active sites that the intermediate occupies and the apparent activation barriers for the redox reaction. In Scheme 1a we show an energy diagram of a metal ion charge transfer reaction involving the adsorbed intermediate written in eqn (1) and (2). We assume that electron transfer is fast so that it occurs concurrently with either adsorption or desorption. This assumption is consistent with observations that rate enhancements arise from changes in the barrier associated with the formation of adsorbed complexes as opposed to the intrinsic barrier for electron transfer.^15,17^ At equilibrium, the reduced and oxidized species are the same energy, and the forward and reverse rates are equal and opposite. The magnitude of these rates is proportional to the experimental rate constant (*k*) and exchange current density (*i*_o_).

The energy of the surface intermediate should correlate with the ease at which the intermediate desorbs from the surface, thus we hypothesize the desorption free energy barrier (Δ*G*^‡^_des_) is a descriptor for redox kinetics. For chemically similar surface reactions, the activation barrier of an elementary step is often linearly correlated to the reaction energy of that step, referred to as a Brønsted–Evans–Polanyi (BEP) relation.^22^ Therefore, the adsorption barrier (Δ*G*^‡^_ads_) linearly correlates with the adsorption energy (Δ*G*_ads_) and the desorption barrier (Δ*G*^‡^_des_) linearly correlates with the desorption energy (Δ*G*_des_) if a BEP relation holds. If true, Δ*G*^‡^_des_ positively correlates with Δ*G*_des_ and negatively correlates with Δ*G*_ads_ (Scheme 1b). The oxidation rate is a function of the concentration of the reduced species ([M^*n*+^]) and the rate constants for adsorption (*k*_ads_) and desorption (*k*_des_) on the surface (derivation in ESI†). Adsorption is rate-limiting at low values of Δ*G*^‡^_des_, whereas desorption is rate-limiting at high values of Δ*G*^‡^_des_, thus the rate is maximized at intermediate Δ*G*^‡^_des_ values at the top of the volcano curve in Scheme 1c.^23^ The rate has contributions from the apparent, or experimentally observed activation energy and frequency factor. The apparent activation energy (Scheme 1d) correlates with Δ*G*^‡^_ads_ (Δ*G*^‡^_des_) when Δ*G*^‡^_des_ is low (high). Similarly, neglecting entropic changes, the apparent frequency factor (Scheme 1e) approaches the frequency factor for the adsorption step, *A*_1_ (desorption step, *A*_2_) at low (high) values of Δ*G*^‡^_des_.

We establish a relationship between desorption free energy barriers of metal–anion complexes and the kinetics of various redox reactions that rationalizes the enhancement by halides following the model discussed in Scheme 1. If BEP relations exist, any of the free energies in Scheme 1 could be calculated, but here we focus on Δ*G*^‡^_des_ due to work showing its relevance to metal ion redox kinetics^24^ and because it can be relatively straightforwardly computed. Using density functional theory (DFT)-based metadynamics simulations, we predict Δ*G*^‡^_des_ of V^3+^–anion complexes on the graphite edge plane (112̄0) [graphite(112̄0)] and compare to our V^2+^/V^3+^ kinetic measurements on edge plane pyrolytic graphite (EPPG) in H_2_SO_4_ and hydrohalic acids. We evaluate V^2+^/V^3+^ experimental exchange current densities (*i*_o_), apparent frequency factors, and apparent activation energies (*E*_a_) on EPPG in sulfuric (H_2_SO_4_), hydrochloric (HCl), and hydriodic (HI) acids, and show that these parameters correlate with the predicted desorption barriers. Desorption barriers of V^3+^–, Cr^3+^–, and Cd^2+^–anion complexes are also calculated on a model mercury Hg(111) electrode to examine whether Δ*G*^‡^_des_ correlates with rate constants across different redox couples and electrodes. These metal ions are chosen because of the availability of experimental rate data displaying an increase in activity in the presence of halides (Fig. 1).

Our results show that desorption barriers of metal–anion complexes on model surfaces correlate with rate constants on polycrystalline electrodes, but inner-sphere electron transfer rates and adsorption of anions are also dependent on the surface structure of the electrode.^25^ When rates of Fe^2+^/Fe^3+^ electron transfer on different facets of Pt^26^ and Au^27^ were examined in non-complexing perchloric acid, the rates correlated with the potential of zero charge (PZC) of the different facets. This finding suggests that the dependence of the rate constant on the local electrode structure comes, at least in part, from variations in the PZC, which affect the structure of the double layer. When Fe^2+^/Fe^3+^ kinetics were measured in sulfuric acid, which is reported to follow an inner-sphere mechanism,^28^ a 30-fold increase in activity was observed at the grain boundaries of a polycrystalline Pt electrode compared to on the grains themselves.^26^ Clearly, the rates for heterogeneous charge transfer reactions are not uniform on a polycrystalline electrode, and corresponding desorption barriers of metal–anion complexes on different facets are likely to change. Although we limit our calculation of desorption barriers to a single facet for different electrodes, we elucidate qualitative trends by correlating desorption barriers of predicted metal–anion surface intermediates to experimental kinetic data on a given electrode surface. Our findings show that desorption barriers of metal–anion surface intermediates are descriptors for redox couple activity across a constant electrode surface and support the hypothesis that halides increase the activity of redox couples by changing the energy and transition states of the adsorbed intermediate.

## Results and discussion

### V^2+^/V^3+^ on graphite edge plane

We study the V^2+^/V^3+^ reaction because its solution-phase structure is known in various electrolytes, and a prior study showed that the desorption barrier of the V^3+^ intermediate in non-complexing solution can be related to redox kinetics.^24^ In HCl, HBr, and HI, V^3+^ complexes with halides to form [V(H_2_O)_5_X]^2+^ (X = Cl, Br, or I).^5,14^ V^3+^ exists predominantly as [V(H_2_O)_5_SO_4_]^+^ in H_2_SO_4_.^5,14^ V^2+^ prefers to form [V(H_2_O)_6_]^2+^ in all the considered electrolytes (*i.e.*, HCl, HBr, HI, and H_2_SO_4_).^5,29^ A metadynamics study predicted the adsorption and desorption barriers of [V(H_2_O)_6_]^2+^ and [V(H_2_O)_6_]^3+^ through an oxygen bridge on graphite(112̄0) and found that [V(H_2_O)_6_]^3+^ desorption had a larger barrier and was rate limiting.^24^ Thus, a lower desorption barrier for the V^3+^-complex should result in faster kinetics.

**Fig. 2:**
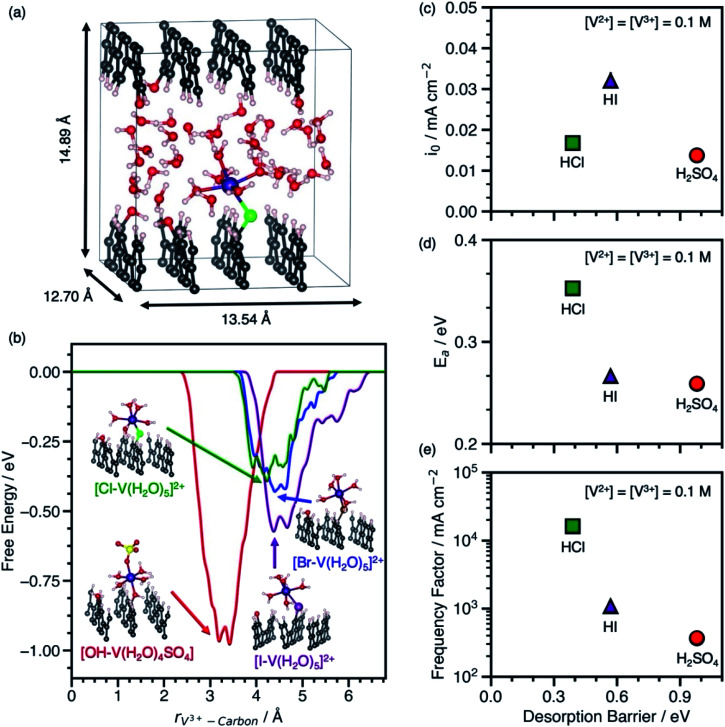
(a) Cell used for metadynamics simulations of V^3+^–anion complexes on graphite(112̄0). (b) Free energy *vs.* the distance between the V^3+^ ion and the carbon surface for V^3+^–anion complexes adsorbed to graphite(112̄0) through an *OH, *Cl, *Br, or *I bridge at 300 K. Geometries of adsorbed V^3+^–anion complexes at the free energy minima are shown next to the corresponding free energy profile in H_2_SO_4_ (red line), HCl (green line), HBr (blue line), and HI (purple line). Atom color legend: C = gray, V = violet, O = red, S = yellow, H = white, Cl = green, Br = dark brown, I = purple. (c) V^2+^/V^3+^ exchange current density (*i*_o_) at room temperature (T = 23.3 °C) on EPPG in H_2_SO_4_, HCl, and HI from steady-state current densities as a function of voltage extrapolated to equilibrium voltage using the Tafel equation *vs.* the V^3+^–anion complex desorption barrier. (d) V^2+^/V^3+^ apparent activation energy (*E*_a_) in H_2_SO_4_, HCl, and HI extracted from the temperature dependence of *i*_o_ from measurements between 23.3−40 °C *vs.* the V^3+^–anion complex desorption barrier. (e) V^2+^/V^3+^ frequency factors *vs.* the V^3+^–anion complex desorption barrier. For all experimental measurements in (c–e), the acid concentration is 1 M and concentrations of both V^2+^ and V^3+^ are 0.1 M.

Our predicted desorption barriers of V^3+^–anion complexes on graphite(112̄0) and experimental kinetic parameters of V^2+^/V^3+^ on EPPG in H_2_SO_4_, HCl, and HI are shown in Fig. 2. The cell and graphite(112̄0) model used in metadynamics simulations to predict Δ*G*^‡^_des_ are shown in Fig. 2a. The EPPG used in experiments consists of multiple parallel edge facets, which resembles graphite(112̄0). The kinetic measurements are conducted at various V^2+^ and V^3+^ concentrations using a rotating disk electrode setup to prevent mass transfer limitations. The change in *E*_a_ at various V^2+^ and V^3+^ concentrations in each electrolyte (Fig. S5 and S6†) indicates that the V^2+^/V^3+^ charge transfer is an inner-sphere reaction that involves an adsorbed intermediate, as opposed to an outer-sphere reaction where *E*_a_ is independent of vanadium concentration. This dependence of apparent activation energy on vanadium concentrations arises because of the dependence of coverage on temperature, which causes the apparent activation energy to include contributions both from the rate constant and enthalpies of adsorption/desorption steps.^14^ The full set of kinetic measurements are provided in Fig. S1–S6, Scheme S1, and Tables S2–S4 and discussed in the ESI.†

The adsorbed [*X–V(H_2_O)_5_] (where X = Cl, Br, or I) and [*OH–V(H_2_O)_4_SO_4_] were used as models for the adsorbed metal–anion complex in hydrohalic acids and H_2_SO_4_, because they preserve the V^3+^ structure in solution and are adsorbed through an anion bridge. The [*OH–V(H_2_O)_4_SO_4_] complex is modeled through an *OH bridge, because *SO_4_ is unstable on carbon surfaces.^14^Fig. 2b shows the desorption free energy profiles of V^3+^–anion complexes from graphite(112̄0) based on metadynamics simulations using spin-polarized DFT with the PBE functional. To calculate Δ*G*^‡^_des_, the distance between the metal ion and the surface was biased until the complex desorbs. Desorption barriers through halide bridges are lower than through an *OH bridge on graphite(112̄0) in the order *OH > *I > *Br > *Cl. Snapshots of desorbed metal–anion complexes are shown in Fig. S7.† Additional DFT and metadynamics modeling details are given in the ESI (Fig. S8–S10 and Table S5†).

The behavior between the measured *i*_o_ in each electrolyte and Δ*G*^‡^_des_ of the V^3+^–anion complexes in Fig. 2c matches the volcano-like relationship in Scheme 1c. V^3+^ adsorbs too strongly in H_2_SO_4_ such that desorption is rate-limiting, and as Δ*G*^‡^_des_ decreases due to weaker V^3+^ adsorption in HI, the rate begins to increase. However, once Δ*G*^‡^_des_ is too low, the adsorption of species become rate-limiting as suggested by rate measurements in HCl. The *i*_o_ has contributions from both *E*_a_ and the apparent frequency factor, which are shown in Fig. 2d and e. The data in Fig. 2d resembles the inverse volcano in Scheme 1d with a hypothetical minimum between [*I–V(H_2_O)_5_]^2+^ in HI and [*OH–V(H_2_O)_4_SO_4_] in H_2_SO_4_. Assuming BEP relations hold, the ideal desorption barrier with the lowest *E*_a_ will be located at that minimum. Despite having the lowest measured *E*_a_ among the electrolytes studied, the V^2+^/V^3+^ exchange current density is lowest in H_2_SO_4_ because it has a low frequency factor, as shown in Fig. 2e. Using this as a model, we predict that the *i*_o_, *E*_a_, and apparent frequency factor of V^2+^/V^3+^ in HBr will be between that in HCl and HI, because the desorption barrier of [*Br–V(H_2_O)_5_]^2+^ is between [*Cl–V(H_2_O)_5_]^2+^ and [*I–V(H_2_O)_5_]^2+^. We also find that the *E*_a_ observed on EPPG in the presence of halides does not correlate with increasing free anion polarizability. This finding highlights the importance of understanding the surface intermediate structure and that using anion physicochemical properties alone are insufficient to explain the observed kinetic behavior.

### V^2+^/V^3+^, Cr^2+^/Cr^3+^, and Cd^0^/Cd^2+^ on mercury

**Fig. 3:**
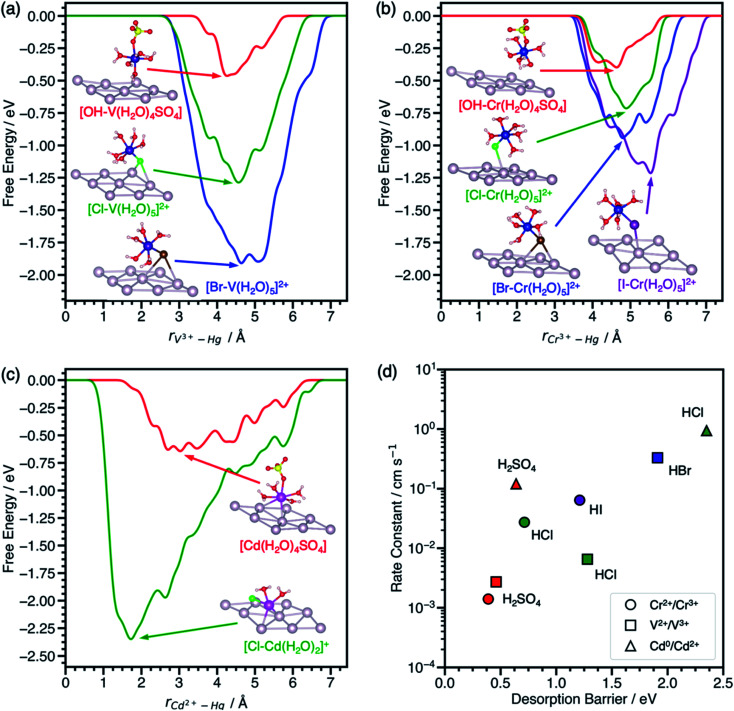
Metadynamics-based free energy profiles for desorption of (a) V^3+^–, (b) Cr^3+^–, and (c) Cd^2+^–anion complexes from Hg(111) through an *OH, *Cl, *Br, or *I bridge at 300 K. Snapshots of the adsorbed metal–anion complexes are shown at the free energy minima. (d) Experimental standard rate constants of the metal charge transfer reactions on mercury drop electrodes plotted against the corresponding predicted desorption barriers from (a–c). Colors denote the acid, namely H_2_SO_4_ (red), HCl (green), HBr (blue), and HI (purple). Rate constants for these reactions were reproduced from ref. 11, 12 and 41. Atom color legend: Hg = gray, V = dark purple, Cr = blue, Cd = pink, O = red, S = yellow, H = white, Cl = green, Br = dark brown, I = purple.

To assess the transferability of the desorption barrier as a descriptor, we compare the Δ*G*^‡^_des_ of V^3+^–, Cr^3+^–, and Cd^2+^–anion complexes on a mercury (Hg) electrode to standard rate constants in sulfuric and hydrohalic acids. Mercury is the most widely reported electrode for anion-promoted electrocatalysis of metal redox couples with several experimental rate constants available in the literature (Table S1†). Anion-promoted electrocatalysis has also been reported on surfaces such as Au, Pt, and carbon, but data is sparse and often consists of only one or two redox couple/electrolyte combinations. We predict the desorption barriers of V^3+^–, Cr^3+^–, and Cd^2+^–anion complexes on a Hg(111) electrode through halide and hydroxyl bridges, Fig. 3. Hg(111) is often used as a model surface (Fig. S8†) for mercury electrodes to study qualitative trends.^30–33^

The effect of halides on the desorption barrier for V^3+^ complexes on Hg(111) are different than on graphite(112̄0). In the case of V^3+^ desorption on Hg(111) (Fig. 3a), [*Br–V(H_2_O)_5_]^2+^ has a desorption barrier of 1.91 eV, followed by [*Cl–V(H_2_O)_5_]^2+^ at 1.28 eV, and [*OH–V(H_2_O)_4_SO_4_] at 0.46 eV. This trend for V^3+^ complexes on Hg(111) is opposite to that of graphite(112̄0), where Δ*G*^‡^_des_ is largest in sulfate electrolytes. This change in trends of desorption barriers for the same redox couple must arise due to the difference in nature of the interaction of the intermediate with Hg(111) and graphite(112̄0). Hg, being noble, has chemisorption that is dominated by Pauli repulsion (especially for electronegative adsorbates such as halides), while chemisorption on graphite is dominated by covalent interactions.^34^ Generally, Δ*G*^‡^_des_ is much larger on Hg(111) than graphite(112̄0).

Cr^3+^ complexes with anions in its first solvation sphere, and charge transfer is predicted to take place through a Cr^3+^–anion bridge on Hg.^15,35^ We predict that Cr^3+^ behaves similarly to V^3+^ on Hg(111), where halides increase the Δ*G*^‡^_des_ relative to sulfate in the same order (*OH < *Cl < *Br < *I), Fig. 3b. The [*OH–Cr(H_2_O)_4_SO_4_] complex only physisorbs on the surface, consistent with experiments suggesting that Cr^2+^/Cr^3+^ charge transfer is outer sphere in the presence of sulfate on Hg electrodes.^8^

We also model the desorption of Cd^2+^ on Hg(111), but only in H_2_SO_4_ and HCl, where rate data for Cd^0^/Cd^2+^ is available.^12^ Cd^2+^–anion complexes have been proposed as the adsorbing species on Hg electrodes in hydrohalic acids.^36,37^ From Fig. 3c, the desorption barrier of the Cd^2+^–chloride complex is 2.35 eV, which is much higher than the Cd^2+^–sulfate complex at 0.64 eV. In the initial geometry, Cd^2+^ was coordinated with five water molecules and one chloride or sulfate ligand, consistent with a report that aqueous Cd^2+^ has a coordination number of six.^38^ During the simulation, Cd^2+^ adsorbed directly onto Hg(111) and formed a [*Cl–Cd(H_2_O)_2_]^+^ complex. After Cd^2+^ desorbed, the solvent waters were reincorporated into the first solvation sphere to form the [Cd(H_2_O)_5_Cl]^+^ complex. The desorption barriers of Cd^2+^ complexes are higher than V^3+^ and Cr^3+^ complexes in the same electrolyte.

The desorption barriers of V^3+^, Cr^3+^, and Cd^2+^ on Hg(111) show a positive correlation with previously measured standard rate constants of the V^2+^/V^3+^, Cr^2+^/Cr^3+^, and Cd^0^/Cd^2+^ reactions on mercury drop electrodes in Fig. 3d. Although Cd^0^/Cd^2+^ metal electrodeposition is expected to follow a different reaction mechanism than the inner-sphere charge transfer of aqueous ions, the correlation still holds. This may occur because the first electron transfer step (*i.e.*, Cd^+^/Cd^2+^) is slower than the solid metal formation (*i.e.*, Cd^0^/Cd^+^), making the kinetic trends more closely resemble that of aqueous charge transfer.^39,40^ This positive correlation between desorption barrier and rate constant suggests that these points are on the left side of the volcano curve in Scheme 1c where adsorption is rate limiting and not around the maximum like V^2+^/V^3+^ on EPPG. For a given redox couple in Fig. 3d, the rate constant and desorption barriers increase going from H_2_SO_4_ (*OH bridge) < HCl (*Cl bridge) < HBr (*Br bridge) < HI (*I bridge). This observation suggests that the more polarizable halides decrease the energy of the active metal–anion intermediate on Hg, thus increasing surface coverage and leading to higher reaction rates. Unlike V^2+^/V^3+^ on EPPG, we do not have experimental apparent activation energies for a more detailed comparison of kinetics on Hg electrodes. We hypothesize that activation energies for V^2+^/V^3+^, Cr^2+^/Cr^3+^, and Cd^0^/Cd^2+^ on Hg will negatively correlate with the desorption barrier and the rate constant.

We also compute desorption barriers of Fe^2+^/Fe^3+^ on Au(111) through *OH, *Cl and *Br bridges (Fig. S11†). Qualitative enhancements in rate constants have been reported for Fe^2+^/Fe^3+^ on gold with increasing concentrations of Cl^−^ and Br^−^ compared to H_2_SO_4_.^10^ The desorption barriers for the [OH–Fe(H_2_O)_4_SO_4_], [Cl–Fe(H_2_O)_5_]^2+^, and [Br–Fe(H_2_O)_5_]^2+^ complexes are predicted to be 0.82, 0.67, and 0.55 eV, respectively, on Au(111). Thus, the rate enhancements in the presence of chloride and bromide may arise due to decreasing the desorption barrier. However, because the reports of enhancements with halides for Fe^2+^/Fe^3+^ on Au(111) are only qualitative, we are unable to obtain quantitative correlations.

The desorption barrier of the electroactive species is a new way to rationalize and predict rate enhancement by halides for inner-sphere aqueous metal ion charge transfer reactions. However, we stress that the desorption barrier is only a valid descriptor among inner-sphere reactions on chemically similar surfaces. When examined together, the rate constants for V^2+^/V^3+^ on Hg(111) and graphite(112̄0) in hydrohalic electrolytes do not correlate in the same way with Δ*G*^‡^_des_ (Fig. S12†). The inability of Δ*G*^‡^_des_ to describe kinetics across different electrodes for a given redox couple can arise due to three major factors: (1) reactions on different surfaces do not necessarily obey the same BEP relations, (2) discrepancies exist between the modeled surface and the actual electrode active site, and (3) different mechanisms occur on different electrodes. By comparing desorption barriers of different metal–anion complexes on the same surface, these factors will be similar and qualitative insights can be gained. Different mechanisms and explanations for anion enhancements may also be valid. Theories have been developed for how electrostatics can be used to explain outer-sphere electron transfer,^42^ and how electrode modification can be used to enhance rates by changing the potential of zero charge.^43,44^ Rate enhancements can also arise from a change in mechanism from outer-sphere to inner-sphere, as is reported to occur for Cr^2+^/Cr^3+^ when halides are added to the electrolyte.^8^ The applicability of using Δ*G*^‡^_des_ as a descriptor for kinetics of the same redox couple on multiple surfaces could be tested by conducting kinetic measurements for a fixed redox couple on chemically similar surfaces such as metal electrodes. Fig. S12,† shows a possible correlation between the desorption barriers for V^3+^ on two noble metals (Au and Hg) and V^2+^/V^3+^ rate constants, but more data is needed for a conclusive analysis. Future work should examine how changes in the desorption barrier on different crystal facets compare to the experimental rate constants on the corresponding single crystal electrodes, which may improve predicted volcano relations and give deeper insight into the structure-sensitivity of inner-sphere metal ion charge transfer.

## Conclusions

This work demonstrates the role of halides in promoting inner-sphere heterogeneous metal charge transfer by changing the transition state energies and energy of the metal–anion surface intermediate. Experimental kinetic measurements combined with metadynamics simulations show that halide bridges increase kinetics of the V^2+^/V^3+^ redox couple on EPPG by decreasing the desorption barrier of the adsorbed V^3+^–anion complex, until an optimum is reached. For V^2+^/V^3+^, Cr^2+^/Cr^3+^, and Cd^0^/Cd^2+^ on Hg, desorption is not rate limiting and halides stabilize the metal ion on the Hg surface, thus increasing surface coverage and promoting the reaction rate. When BEP relations hold for chemically similar reactions, desorption barriers correlate with redox kinetics. This knowledge can guide anion bridge design so reactive intermediates adsorb on electrodes with optimal strength. Because the charge transfer kinetics of many metal ion redox couples are increased in the presence of halides, these findings apply broadly and highlight the importance of understanding the combined role of the redox couple, electrode, and electrolyte when engineering electrochemical systems.

## Data availability

Data for this paper, including VASP output files and geometries, are available at the NOMAD Repository at https://dx.doi.org/10.17172/NOMAD/2021.07.03-1.

## Author contributions

B. R. G. and N. S. conceived the project. J. F. performed all the molecular simulations and DFT modeling under the guidance of B. R. G. H. A. carried out all electrochemical measurements and data analysis under the guidance of N. S. All authors were involved in analysis and writing the manuscript.

## Conflicts of interest

There are no conflicts to declare.

## Supplementary Material

SC-012-D1SC03642D-s001
