# Supplementary material for: Why halides enhance heterogeneous metal ion charge transfer reactions
Source: Chem Sci. 2021 Aug 26;12(38):12704–10. doi: 10.1039/d1sc03642d (PMC8494035; doi:10.1039/d1sc03642d)
Supplement: SC-012-D1SC03642D-s001 [file SC-012-D1SC03642D-s001.pdf]

Electronic Supplementary Material (ESI) for Chemical Science.
This journal is © The Royal Society of Chemistry 2021

SUPPORTING INFORMATION

Why Halides Enhance Heterogeneous Metal Ion Charge Transfer Reactions

Jacob Florian, Harsh Agarwal, Nirala Singh* and Bryan R. Goldsmith*

Department of Chemical Engineering, University of Michigan, Ann Arbor, Michigan 48109-2136, United States

Catalysis Science and Technology Institute, University of Michigan, Ann Arbor, Michigan 48109-2136, United States

S1. Rate Constants for Metal Ion Redox Couples

Table S1. Standard rate constants for metal ion redox couples with and without halide anions.

**Redox Couple**	**Electrode***	**Electrolyte****	**Rate Constant / cm s^{-1}**	**Reference**
Ag^{+}/Ag^{0}	Ag	$HClO_4$	5.18×10^{-3}	[1]
Bi^{3+}/Bi^{0}	Bi_{Hg}	$HClO_4$ *(ref)*	3.70×10^{-4}	[2]
Bi^{3+}/Bi^{0}	Bi_{Hg}	H_2SO_4	1.80×10^{-3}	[2]
Bi^{3+}/Bi^{0}	Bi_{Hg}	$HClO_4 + Cl^{-}$	2.80×10^{-3}	[2]
Bi^{3+}/Bi^{0}	Bi_{Hg}	$HClO_4 + I^{-}$	2.90×10^{-1}	[3]
Bi^{3+}/Bi^{0}	Hg	$HClO_4$	3.70×10^{-4}	[2]
Bi^{3+}/Bi^{0}	Hg	H_2SO_4	1.85×10^{-3}	[2]
Bi^{3+}/Bi^{0}	Hg	HCl	2.70×10^{-3}	[2]
Cd^{2+}/Cd^{0}	Cd_{Hg}	$HClO_4$	3.52×10^{-1}	[4]
Cd^{2+}/Cd^{0}	Cd_{Hg}	$NaClO_4$ *(ref)*	5.00×10^{-1}	[5]
Cd^{2+}/Cd^{0}	Cd_{Hg}	Na_2SO_4	5.50×10^{-1}	[5]
Cd^{2+}/Cd^{0}	Cd_{Hg}	KCl	1.25×10^{0}	[6]
Cd^{2+}/Cd^{0}	Cd_{Hg}	KBr	9.10×10^{-1}	[6]
Cd^{2+}/Cd^{0}	Cd_{Hg}	KI	6.50×10^{0}	[6]
Cd^{2+}/Cd^{0}	Hg	$HClO_4$ *(ref)*	2.50×10^{-1}	[7]
Cd^{2+}/Cd^{0}	Hg	H_2SO_4	1.20×10^{-1}	[7]
Cd^{2+}/Cd^{0}	Hg	HCl	9.40×10^{-1}	[7]
Ce^{4+}/Ce^{3+}	Au	$HClO_4$	4.70×10^{-5}	[8]
Ce^{4+}/Ce^{3+}	Pt	H_2SO_4	3.70×10^{-4}	[9]
Co^{2+}/Co^{0}	Co	$CoSO_4$ *(ref)*	6.74×10^{-8}	[10]
Co^{2+}/Co^{0}	Co	$CoSO_4 + Cl^{-}$	5.70×10^{-9}	[10]
Co^{2+}/Co^{0}	Co	$CoSO_4 + I^{-}$	5.18×10^{-10}	[10]
Cr^{2+}/Cr^{3+}	Hg	$HClO_4$ *(ref)*	1.40×10^{-3}	[11]
Cr^{2+}/Cr^{3+}	Hg	$HClO_4 + Cl^{-}$	2.73×10^{-2}	[11]
Cr^{2+}/Cr^{3+}	Hg	$HClO_4 + I^{-}$	6.40×10^{-2}	[11]
Cr^{3+}/Cr^{2+}	Hg	$NaClO_4$	8.00×10^{-6}	[12]
Cr^{3+}/Cr^{2+}	Hg	KCl	8.50×10^{-6}	[12]
Cr^{3+}/Cr^{2+}	Hg	KBr	1.70×10^{-5}	[12]
Cr^{3+}/Cr^{2+}	Hg	KI	9.80×10^{-5}	[12]
Cu^{2+}/Cu^{0}	Cu	H_2SO_4	4.20×10^{-4}	[13]
Eu^{2+}/Eu^{3+}	Hg	$HClO_4$ *(ref)*	1.70×10^{-4}	[14]
Eu^{2+}/Eu^{3+}	Hg	HCl	2.40×10^{-4}	[14]
Eu^{2+}/Eu^{3+}	Hg	HBr	9.80×10^{-4}	[14]
Fe^{2+}/Fe^{3+}	Au	$HClO_4$	8.00×10^{-5}	[15]
Fe^{2+}/Fe^{3+}	Au	H_2SO_4 *(ref)*	1.00×10^{-2}	[16,17]
Fe^{2+}/Fe^{3+}	Au	HCl	3.10×10^{-2}	[17]
Fe^{2+}/Fe^{3+}	Pt	H_2SO_4 *(ref)*	1.30×10^{-3}	[18]
Fe^{2+}/Fe^{3+}	Pt	HCl	4.40×10^{-3}	[18]

Fe^{2+}/Fe^{3+}	Ru	H_2SO_4	7.83×10^{-4}	19
Fe^{2+}/Fe^{3+}	Glassy Carbon	$HClO_4$	1.34×10^{-3}	20
Fe^{2+}/Fe^{3+}	Glassy Carbon	H_2SO_4 *(ref)*	1.20×10^{-3}	20
Fe^{2+}/Fe^{3+}	Glassy Carbon	HCl	5.70×10^{-3}	20
Fe^{3+}/Fe^{2+}	Rh	$HClO_4$	4.60×10^{-4}	21
Fe^{3+}/Fe^{2+}	Rh	H_2SO_4	2.28×10^{-3}	22
In^{3+}/In^0	In_{Hg}	$HClO_4 + SO_4^{2-}$ *(ref)*	5.87×10^{-5}	23
In^{3+}/In^0	In_{Hg}	$HClO_4 + Cl^-$	5.87×10^{-5}	23
In^{3+}/In^0	In_{Hg}	$HClO_4 + Br^-$	1.00×10^{-3}	23
In^{3+}/In^0	In_{Hg}	$HClO_4 + I^-$	3.01×10^{-3}	23
In^{3+}/In^0	Hg	$NaClO_4$ *(ref)*	1.28×10^{-3}	24
In^{3+}/In^0	Hg	NaCl	3.40×10^{-2}	25
In^{3+}/In^0	Hg	NaBr	6.50×10^{-1}	25
Ni^{2+}/Ni^0	Ni	H_2SO_4 *(ref)*	8.60×10^{-10}	26
Ni^{2+}/Ni^0	Ni	HCl	5.70×10^{-9}	26
Pb^{2+}/Pb^0	Pb_{Hg}	$HClO_4$	1.00×10^{0}	27
Pb^{2+}/Pb^0	Pb_{Hg}	$NaClO_4$ *(ref)*	1.02×10^{1}	6
Pb^{2+}/Pb^0	Pb_{Hg}	KCl	5.20×10^{0}	6
Pb^{2+}/Pb^0	Pb_{Hg}	KBr	9.60×10^{-1}	6
Pb^{2+}/Pb^0	Hg	NaCl	1.80×10^{-1}	25
Pb^{2+}/Pb^0	Hg	NaBr	4.80×10^{-1}	25
Pd^{2+}/Pd^0	Pd	HCl	2.49×10^{-6}	28
Pd^{2+}/Pd^0	Pd	HBr	4.04×10^{-6}	28
Pt^{4+}/Pt^{2+}	Pt	HCl	2.30×10^{-7}	29
Pt^{4+}/Pt^{2+}	Pt	HBr	6.30×10^{-6}	29
Pt^{4+}/Pt^{2+}	Pt	HI	7.20×10^{-5}	29
Tl^{+}/Tl^0	Tl_{Hg}	$NaClO_4$ *(ref)*	3.40×10^{0}	6
Tl^{+}/Tl^0	Tl_{Hg}	K_2SO_4	3.50×10^{0}	6
Tl^{+}/Tl^0	Tl_{Hg}	KCl	5.00×10^{0}	6
V^{2+}/V^{3+}	Hg	$HClO_4$ *(ref)*	1.10×10^{-3}	14
V^{2+}/V^{3+}	Hg	H_2SO_4	2.70×10^{-3}	14
V^{2+}/V^{3+}	Hg	HCl	6.50×10^{-3}	14
V^{2+}/V^{3+}	Hg	HBr	3.30×10^{-1}	14
V^{2+}/V^{3+}	Glassy Carbon	H_2SO_4 *(ref)*	2.70×10^{-8}	30
V^{2+}/V^{3+}	Glassy Carbon	HCl	6.73×10^{-8}	30
V^{2+}/V^{3+}	Glassy Carbon	HBr	1.16×10^{-7}	30
V^{2+}/V^{3+}	Glassy Carbon	HI	2.19×10^{-7}	30
Zn^{2+}/Zn^0	Zn_{Hg}	$NaClO_4$ *(ref)*	2.48×10^{-3}	31
Zn^{2+}/Zn^0	Zn_{Hg}	KCl	5.05×10^{-3}	31
Zn^{2+}/Zn^0	Zn_{Hg}	NaBr	1.03×10^{-2}	31
Zn^{2+}/Zn^0	Zn_{Hg}	NaI	1.31×10^{-1}	31
Zn^{2+}/Zn^0	Hg	K_2SO_4 *(ref)*	1.30×10^{-3}	32
Zn^{2+}/Zn^0	Hg	KCl	4.00×10^{-3}	33
Zn^{2+}/Zn^0	Hg	KBr	8.00×10^{-3}	33
Zn^{2+}/Zn^0	Hg	KI	7.00×10^{-2}	33

*$Metal_{Hg}$ electrodes are metal-Hg amalgam alloy electrodes. ***(ref)* indicates reference used for $k_{no\ halides}$ in **Figure 1**. The *ref* is chosen as a perchlorate- or sulfate-based salt solution (instead of acid) for the cases where the rate data in presence of halides is available only in halide-based salt solutions to eliminate the effect of pH on rate constants.

Rate constants in **Table S1** are calculated at room temperature using various methods. For comparing rate data to desorption barriers in **Figure 3**, rate constants in hydrohalic and sulfuric acid electrolytes were taken from a single reference for each redox couple to avoid discrepancies in the experimental method used.

S2. Derivation of Rate Law

For deriving the rate law in **Scheme 1** of the main text, the conversion between M^{n+} and M^{n+1} was considered to occur through an adsorbed surface intermediate $* XM^{n+1}$.

$$* X + M^{n+} \rightleftarrows * XM^{n+1} + e^- \rightleftarrows * X + M^{n+1} + e^- \quad \textbf{(S1)}$$

Because the concentration of the adsorbed intermediate is much lower than the bulk concentrations of the ions in solution, the overall rate of formation of the surface intermediate ($r_{*XM^{n+1}}$) is assumed to be zero (i.e., the pseudo-steady-state hypothesis). Only the forward rates are considered for this model, given that the exchange current density is proportional to the forward rate at equilibrium.

$$r_{*XM^{n+1}} = k_{ads}\theta_X[M^{n+}] - k_{des}\theta_{*XM^{n+1}} = 0 \quad \textbf{(S2)}$$

This gives an expression for the coverage of the surface intermediate ($\theta_{*XM^{n+1}}$).

$$\theta_{*XM^{n+1}} = \frac{k_{ads}\theta_X[M^{n+}]}{k_{des}} \quad \textbf{(S3)}$$

$$\theta_{*XM^{n+1}} + \theta_X = 1 \quad \textbf{(S4)}$$

The forward rate law can be expressed in terms of the coverage of the surface intermediate. The units of $k_{ads}[\frac{1}{s}]$ and $k_{des}[\frac{mol}{L \cdot s}]$ in the elementary rate expression are not the same because the rate of adsorption is proportional to the concentration of M^{n+} and the coverage of adsorbed halides (dimensionless), whereas the rate of desorption is proportional to the coverage of the intermediate (dimensionless). By using coverages instead of concentrations of adsorbed species, the rate is normalized to the total number of active sites on the electrode surface.

$$r = k_{des}\theta_{*XM^{n+1}} \quad \textbf{(S5)}$$

$$r = \frac{k_{ads}k_{des}[M^{n+}]}{k_{des} + k_{ads}[M^{n+}]} \quad \textbf{(S6)}$$

The values of k_{ads} and k_{des} are a function of the adsorption barrier ($\Delta G^{\ddagger}_{ads}$) and desorption barrier ($\Delta G^{\ddagger}_{des}$). For chemically similar reactions, $\Delta G^{\ddagger}_{ads}$ and $\Delta G^{\ddagger}_{des}$ can be linearly correlated to the adsorption (ΔG_{ads}) and desorption (ΔG_{des}) free energies through Brønsted-Evans-Polanyi (BEP) relations.

$$\Delta G^{\ddagger}_{ads} = \Delta G^{\ddagger}_{ads,0} + \beta_1 \Delta G_{ads} \quad \textbf{(S7)}$$

$$\Delta G^{\ddagger}_{des} = \Delta G^{\ddagger}_{des,0} + \beta_2 \Delta G_{des} \quad \textbf{(S8)}$$

Here β_{i} describes the position of the transition state along the reaction coordinate, and $\Delta G^{\ddagger}_{ads,0}$ ($\Delta G^{\ddagger}_{des,0}$) is a constant that represents the transition state energy for adsorption (desorption) when $\Delta G_{ads} = 0$ ($\Delta G_{des} = 0$). Further, by assuming that the electron transfer step is fast and there is only one adsorbed intermediate, the adsorption and desorption free energies are equal and opposite ($\Delta G_{ads} = -\Delta G_{des}$). Thus, $\Delta G^{\ddagger}_{ads}$ is linearly related to $\Delta G^{\ddagger}_{des}$.

$$\Delta G^{\ddagger}_{ads} = \Delta G^{\ddagger}_{ads,0} + \beta_1 \Delta G_{ads} = \Delta G^{\ddagger}_{ads,0} + \beta_1(-\Delta G_{des}) = \Delta G^{\ddagger}_{ads,0} - \beta_1 \left(\frac{\Delta G^{\ddagger}_{des} - \Delta G^{\ddagger}_{des,0}}{\beta_2} \right) \quad \textbf{(S9)}$$

With this, the forward rate in **Eq. S6** can be defined in terms of $\Delta G^{\ddagger}_{des}$, $[M^{n+}]$, and constants $\Delta G^{\ddagger}_{ads,0}$, $\Delta G^{\ddagger}_{des,0}$, β_1, β_2, A_1, and A_2.

$$r=\frac{A_1\exp\left(-\frac{\Delta G^{\ddagger}_{ads}}{RT}\right)A_2\exp\left(-\frac{\Delta G^{\ddagger}_{des}}{RT}\right)[M^{n+}]}{A_2\exp\left(-\frac{\Delta G^{\ddagger}_{des}}{RT}\right)+A_1\exp\left(-\frac{\Delta G^{\ddagger}_{ads}}{RT}\right)[M^{n+}]}=\frac{A_1\exp\left(-\frac{\Delta G^{\ddagger}_{ads,0}-\beta_1\left(\frac{\Delta G^{\ddagger}_{des}-\Delta G^{\ddagger}_{des,0}}{\beta_2}\right)}{RT}\right)A_2\exp\left(-\frac{\Delta G^{\ddagger}_{des}}{RT}\right)[M^{n+}]}{A_2\exp\left(-\frac{\Delta G^{\ddagger}_{des}}{RT}\right)+A_1\exp\left(-\frac{\Delta G^{\ddagger}_{ads,0}-\beta_1\left(\frac{\Delta G^{\ddagger}_{des}-\Delta G^{\ddagger}_{des,0}}{\beta_2}\right)}{RT}\right)[M^{n+}]} \quad \textbf{(S10)}$$

Eq. S10 was used to generate the plot of the rate in **Scheme 1**. The derivative of the log of the rate with respect to inverse temperature was used to extract the apparent activation energy and the apparent frequency factor.

S3. Experimental V^{2+}/V^{3+} Kinetic Measurements in H_2SO_4, HCl, and HI on EPPG

V^{2+}/V^{3+} kinetic measurements were conducted on Edge Plane Pyrolytic Graphite (EPPG) (3.81 mm outer diameter × 4 mm thick, Pine Research) using the experimental setup described in prior work.[30,34] The EPPG disk was encapsulated in epoxy for stability, making the total outer diameter of the disk 5 mm. A three-electrode two-compartment cell, with the two compartments separated by a Nafion 117 membrane (Fuel Cell store), was used. A double junction Ag/AgCl (Pine Research) was used as the reference electrode, and a graphite rod (99.9995 % metals basis, Alfa Aesar) was used as the counter electrode. The reference electrode was calibrated against the reversible hydrogen electrode by comparing to a Pt wire in the supporting electrolyte with 1 bar H_2 bubbled into solution. Vanadium(IV) sulfate hydrate ($VOSO_4{\cdot}xH_2O$ [*x* assumed to be 5], ≥ 99.99% trace metals basis, Sigma Aldrich) in sulfuric acid (H_2SO_4, 99.999%, Sigma Aldrich), vanadium (III) chloride (VCl_3, 99% metals basis, Alfa Aesar) in hydrochloric acid (HCl, ACS Reagent 37%, Sigma Aldrich), and vanadium (V) oxide (V_2O_5, ≥ 99.6% trace metals basis, Sigma Aldrich) in hydriodic acid (HI, 57%, contains no stabilizer, Sigma Aldrich) was used as the starting salt for preparing electrolytes to avoid the presence of any other anions in solution and isolate the effect of the specific anion on reaction kinetics. All solutions were prepared using purified water with 18.2 MΩ cm resistivity obtained from Millipore Sigma Synergy Ultrapure Water Purification System. The concentration of vanadium ions in each prepared electrolyte was 0.2 M and the acid concentration was fixed at 1 M.

The electrolytes were prepared by the pre-electrolysis method described in our previous work.[30,34] The prepared solutions were first reduced to V^{2+}, followed by oxidation to reach the specific concentration distribution of V^{2+} and V^{3+}. The concentrations of both V^{2+} and V^{3+} were confirmed via UV-Vis spectroscopy, using the calibration curves and Gaussian fitting parameters available in our prior work.[30,34]

H_2SO_4 was used as the counter electrolyte solution during pre-electrolysis for measurements in H_2SO_4. However, for measurements in HCl and HI, perchloric acid ($HClO_4$, 60%, Fisher Chemical) was used as the counter electrolyte solution, which was replaced after pre-electrolysis to prevent the contribution of currents due to formed Cl_2 or I_2 by the crossover of Cl^-/I^- anions from the working electrolyte compartment.[30] Further, H_2SO_4 was not used as the counter electrolyte solution for measurements in HCl and HI because of the inhibiting effect of sulfate on the measured V^{2+}/V^{3+} kinetics in HI on a glassy carbon electrode.[30,34] ClO_4^- being a non-interacting anion ensures that the nature of the intermediate is unaffected in HCl and HI.

All electrochemical experiments were conducted on a cleaned EPPG electrode (cleaning process of EPPG described below) using a VSP potentiostat/galvanostat with a built-in electrochemical impedance spectroscopy (EIS) analyzer (Biologic Science Instruments USA). The exchange current densities (i_o) were extracted by conducting steady state ($i_{o,Tafel}$) and EIS ($i_{o,R_{ct}}$) measurements described below. The two independent techniques were in good agreement, providing additional confidence in the reproducibility of our kinetic measurements. The apparent activation energies (E_a) were extracted from the temperature dependence of i_o. Temperature was controlled by a refrigerated/heated bath circulator (Fisher Scientific).

Steady state current measurements at various rotation rates: Steady state measurements were conducted between +300 mV and −100 mV overvoltage for different combinations of V^{2+} and V^{3+} concentrations at various rotations rates to confirm the measurements are in the kinetic regime. If measurements were not in the kinetic regime for highly oxidative overpotentials, the kinetic currents were evaluated by Koutecky-Levich analysis.[35] These kinetic currents were normalized by the electrochemical active surface area (ECSA) and plotted in the log scale vs. the applied overvoltage to obtain the Tafel plot. The log of oxidative kinetic currents in the region of 117−300 mV were extrapolated to zero overvoltage and the intercept was used to obtain $i_{o,Tafel}$.

Electrochemical Impedance Spectroscopy: EIS measurements were conducted at open circuit voltage (OCV) with an overlaid 10 mV amplitude sine wave for a frequency range of 500 kHz to 100 mHz (with six points per decade) at each desired combination of V^{2+} and V^{3+} concentrations. The EIS measurements were taken before beginning each set of steady state measurement at a particular rotation rate. Before each EIS measurement, the OCV was equilibrated for 15 seconds. The measurements were fitted to a modified Randles circuit.[35] The charge transfer resistance (R_{ct}) values were used to evaluate $i_{o,R_{ct}}$ at each rotation rate, which were then averaged and used for analysis. The change in OCV among different electrolytes (**Figure S1**) can be potentially caused by the difference in the stability of V^{3+} complexes formed in H_2SO_4, HCl and HI. It has been shown previously that V^{3+} complexes differently in each of these electrolytes.[30] Tafel and Nyquist plots from the EIS data, and the measurements to extract the capacitance to estimate the electrochemical active surface area in H_2SO_4 (**Figure S2**, **Table S2**), HCl (**Figure S3**, **Table S3**), and HI (**Figure S4**, **Table S4**) are given below.

Apparent activation energies: $E_{a,Tafel}$ and $E_{a,R_{ct}}$ for each combination of V^{2+} and V^{3+} concentrations were evaluated by using the temperature variation (at 23.3, 30, 35, and 40 °C) of the corresponding $i_{o,Tafel}$ and $i_{o,R_{ct}}$ values, respectively, and using an Arrhenius relationship, as done in our prior work.[30,34]

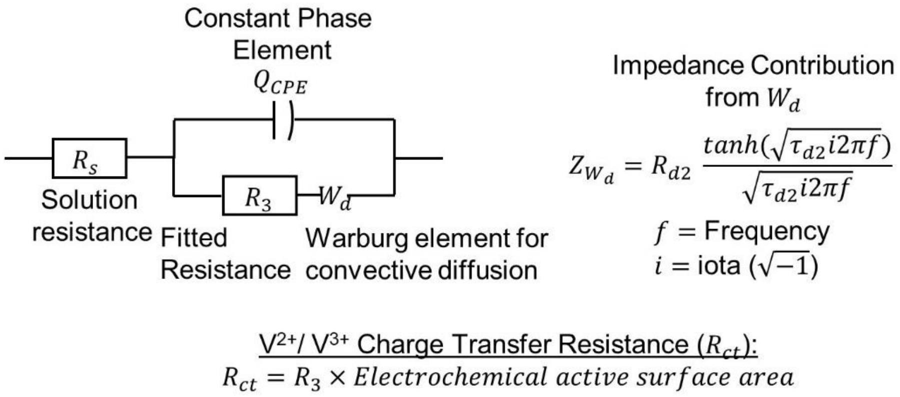

Scheme S1. Modified Randles circuit used for fitting all the EIS measurements for V^{2+}/V^{3+} reaction in different electrolytes. R_s is the solution resistance, Q_{CPE} is the constant phase element, R_{ct} is the charge transfer resistance for V^{2+}/V^{3+} redox reaction obtained by multiplying fitted R_3 with the electrochemically active surface area, and W_d is the Warburg element for convective diffusion to account for the mass transfer at low frequencies.

Impedance from W_d (Z_{W_d}) is a function of resistance R_{d2} and time constant t_{d2}. R_s, Q_{CPE}, R_3, R_{d2}, and t_{d2} are obtained by the Zfit application of EC-Lab Software V11.18.[36]

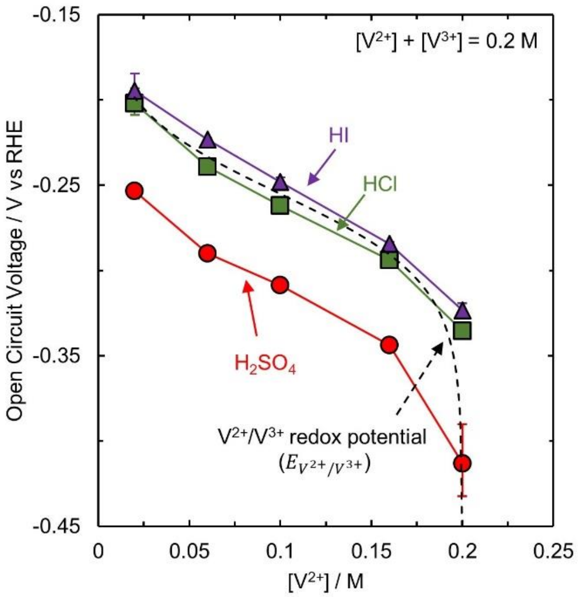

Figure S1. Open Circuit Voltage vs RHE at different $[V^{2+}]$ with total [V] = 0.2 M at room temperature (23.3 ° C) in different tested electrolytes. The V^{2+}/V^{3+} redox potential obtained from the Nernst equation for a total [V] = 0.2 M is shown by the dotted line. The standard redox potential of V^{2+}/V^{3+} ($E^o_{V^{2+}/V^{3+}}$) is −0.255 V (vs RHE).

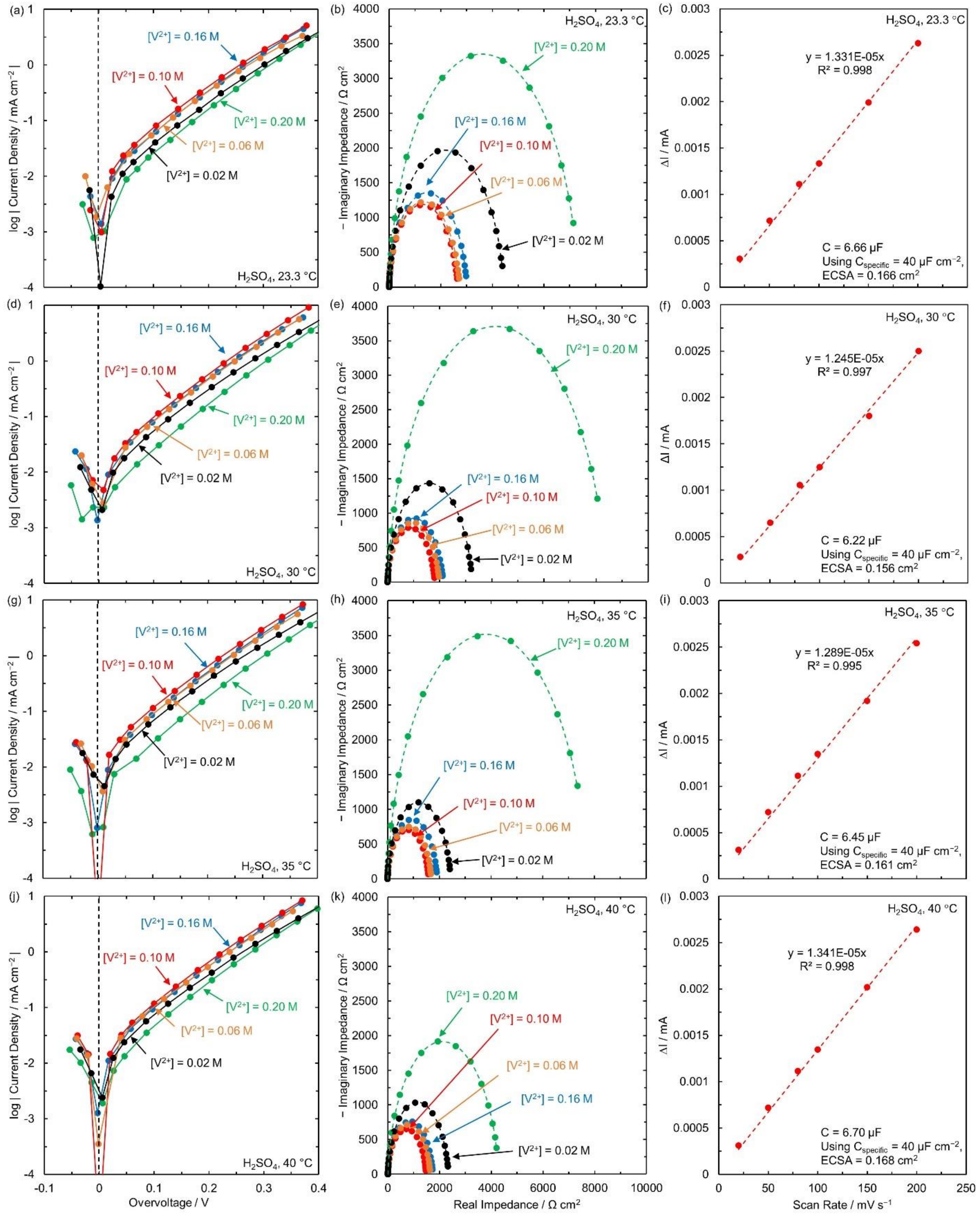

Figure S2. Tafel plots (a, d, g, j) and Nyquist plots (b, e, h, k) at various [V^{2+}] concentrations (0.20, 0.16. 0.10, 0.06, and 0.02 M) at different temperatures (23.3, 30, 35, and 40 °C) in H_2SO_4 on edge plane pyrolytic graphite electrode. Tafel plots are constructed from steady-state measurements corrected for solution resistance. Circles in Tafel plots correspond to experimental measured points while the lines are used to guide the eye. The circles in the Nyquist plots are from EIS experimental measurements (b, e, h, k) and the dotted lines represent fits obtained from the EC-Lab software after modelling as a modified Randles circuit. All fitting parameters are available in Table S2. The ECSA (c,

f, i, l) was obtained by the cyclic voltammetry capacitance method in H_2SO_4 for measurements in H_2SO_4. Note that all these plots are from a single experimental run. Each condition was repeated three times and the average exchange current densities evaluated from the three runs are reported in the main text.

Table S2. Fitted parameters obtained for a single experimental run using the Zfit application in EC-Lab software for H_2SO_4 at various [V^{2+}] concentrations at different temperatures on edge plane pyrolytic graphite electrode. A modified Randles circuit is used for fitting all the EIS measurements. The obtained fits are shown by dotted lines in Nyquist plots and the ECSA is evaluated from cyclic voltammetry capacitance method shown in Figure S2. Each condition was repeated three times and the average exchange current densities evaluated from the three runs are reported.

**H_2SO_4**									
T (°C)	[V^{2+}] (M)	R_s (Ω)	Q_{CPE} (F $s^{(a-1)}$)	a	R_{d2} (Ω)	t_{d2} (s)	R_3 (Ω)	ECSA (cm^2)	R_{ct} (Ω cm^2)
23.3	0.20	5.84	4.44×10^{-6}	0.955	1020	1.197	42900	0.166	7121.4
	0.16	5.91	4.16×10^{-6}	0.953	538	0.661	17400		2888.4
	0.10	6.62	3.99×10^{-6}	0.953	583	0.645	15300		2539.8
	0.06	8.18	3.87×10^{-6}	0.954	560	0.686	15800		2622.8
	0.02	7.06	3.75×10^{-6}	0.943	794	0.847	25800		4282.8
30.0	0.20	5.34	4.16×10^{-6}	0.935	1300	1.816	52600	0.156	8205.6
	0.16	5.23	3.97×10^{-6}	0.944	712	0.491	12900		2012.4
	0.10	4.55	3.99×10^{-6}	0.944	561	0.526	11000		1716.0
	0.06	5.24	4.01×10^{-6}	0.943	559	0.544	12100		1887.6
	0.02	4.98	4.10×10^{-6}	0.939	499	0.825	20300		3166.8
35.0	0.20	5.49	5.77×10^{-6}	0.948	5900	146.7	47300	0.161	7615.3
	0.16	5.82	5.50×10^{-6}	0.954	577	0.487	11300		1819.3
	0.10	6.08	5.46×10^{-6}	0.954	475	0.487	9310		1498.9
	0.06	6.27	5.51×10^{-6}	0.951	433	0.492	9970		1605.2
	0.02	6.64	5.48×10^{-6}	0.952	429	0.692	14600		2350.6
40.0	0.20	8.26	5.25×10^{-6}	0.942	571	0.957	24900	0.168	4183.2
	0.16	8.09	4.91×10^{-6}	0.947	502	0.407	9800		1646.4
	0.10	8.37	4.77×10^{-6}	0.947	381	0.472	8410		1412.9
	0.06	7.72	4.62×10^{-6}	0.947	439	0.415	9290		1560.7
	0.02	7.66	4.57×10^{-6}	0.947	554	0.539	13400		2251.2

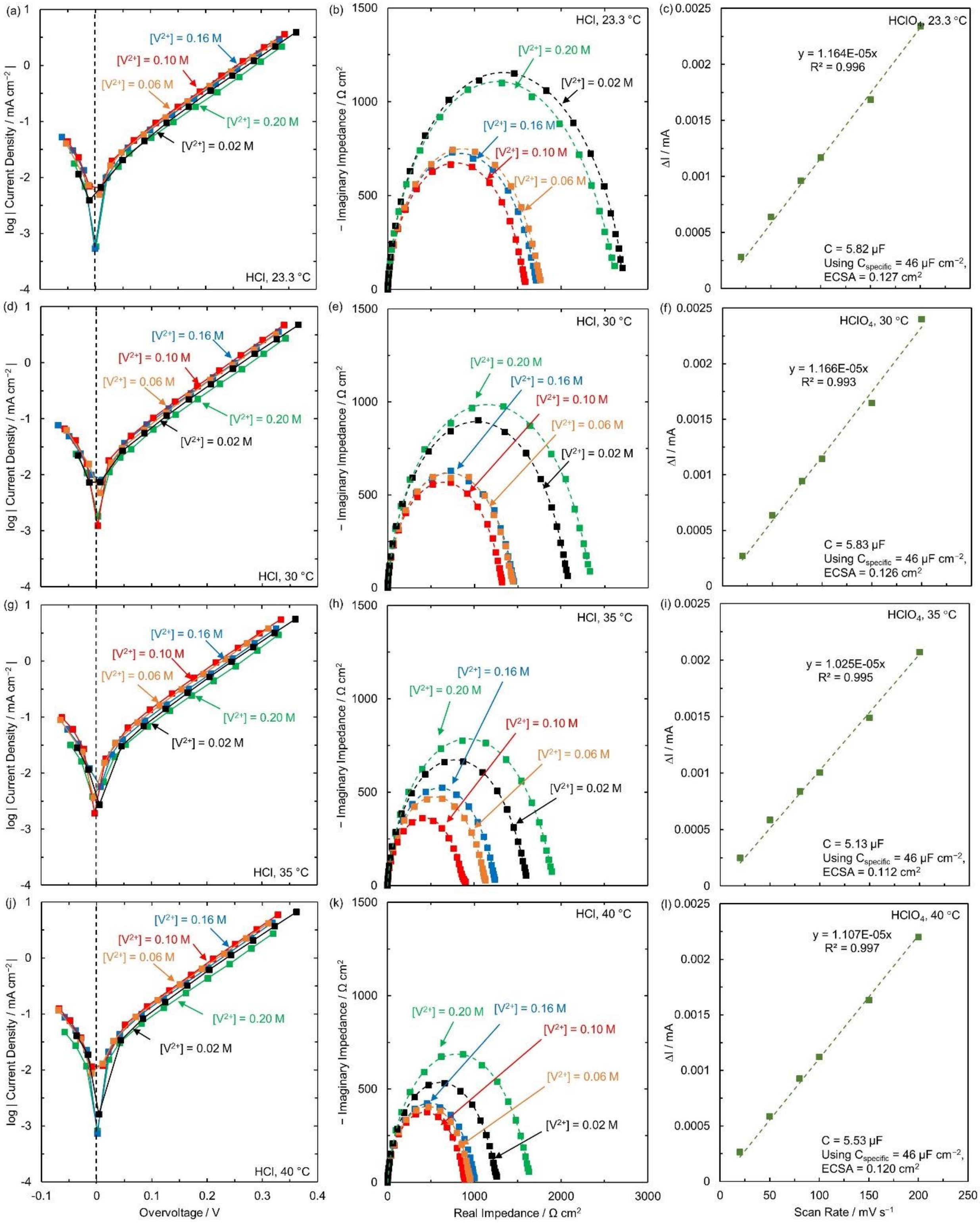

Figure S3. Tafel plots (a, d, g, j) and Nyquist plots (b, e, h, k) at various [V^{2+}] concentrations (0.20, 0.16. 0.10, 0.06, and 0.02 M) at different temperatures (23.3, 30, 35, and 40 °C) in HCl on edge plane pyrolytic graphite electrode. Tafel plots are constructed from steady-state measurements corrected for solution resistance. Squares in Tafel plots correspond to experimental measured points while the lines are used to guide the eye. The squares in the Nyquist plots are from EIS experimental measurements (b, e, h, k) and the dotted lines represent fits obtained from the EC-Lab

software after modelling as a modified Randles circuit. All fitting parameters are available in Table S3. The ECSA (c, f, i, l) was obtained by the cyclic voltammetry capacitance method in $HClO_4$ for measurements in HCl. Note that all these plots are from a single experimental run. Each condition was repeated three times and the average exchange current densities evaluated from the three runs are reported in the main text.

Table S3. Fitted parameters obtained for a single experimental run using the Zfit application in EC-Lab software for HCl at various [V^{2+}] concentrations at different temperatures on edge plane pyrolytic graphite electrode. A modified Randles circuit is used for fitting all the EIS measurements. The obtained fits are shown by dotted lines in Nyquist plots and the ECSA is evaluated from cyclic voltammetry capacitance method shown in Figure S3. Each condition was repeated three times and the average exchange current densities evaluated from the three runs are reported.

HCl									
T (°C)	[V^{2+}] (M)	R_s (Ω)	Q_{CPE} (F $s^{(a-1)}$)	a	R_{d2} (Ω)	t_{d2} (s)	R_3 (Ω)	ECSA (cm^2)	R_{ct} (Ω cm^2)
23.3	0.20	13.6	3.04×10^{-6}	0.916	784	0.924	19900	0.127	2527.3
	0.16	16.5	2.76×10^{-6}	0.912	396	0.492	13100		1663.7
	0.10	15.5	2.78×10^{-6}	0.909	258	0.839	12300		1562.1
	0.06	16.8	2.74×10^{-6}	0.911	301	0.803	13600		1727.2
	0.02	15.5	2.67×10^{-6}	0.917	808	0.439	20600		2616.2
30.0	0.20	4.89	3.00×10^{-6}	0.904	350	1.003	18100	0.126	2280.6
	0.16	4.78	2.68×10^{-6}	0.920	436	0.313	11000		1386.0
	0.10	5.58	2.60×10^{-6}	0.925	336	0.439	10000		1260.0
	0.06	5.01	2.73×10^{-6}	0.914	232	0.951	11100		1398.6
	0.02	5.05	2.70×10^{-6}	0.918	325	1.096	16000		2016.0
35.0	0.20	10.2	3.06×10^{-6}	0.898	565	0.710	16500	0.112	1848.0
	0.16	4.95	2.81×10^{-6}	0.912	399	0.498	10700		1198.4
	0.10	4.47	3.26×10^{-6}	0.900	372	0.746	7590		850.1
	0.06	8.42	2.73×10^{-6}	0.913	394	0.511	9720		1088.6
	0.02	8.93	2.80×10^{-6}	0.909	450	0.688	13760		1541.1
40.0	0.20	7.12	3.14×10^{-6}	0.908	424	0.699	13100	0.120	1572.0
	0.16	7.58	2.96×10^{-6}	0.913	259	0.430	8050		966.0
	0.10	4.24	2.84×10^{-6}	0.918	241	0.431	7140		856.8
	0.06	4.26	2.77×10^{-6}	0.919	245	0.472	7650		918.0
	0.02	5.16	2.76×10^{-6}	0.920	370	0.532	10100		1212.0

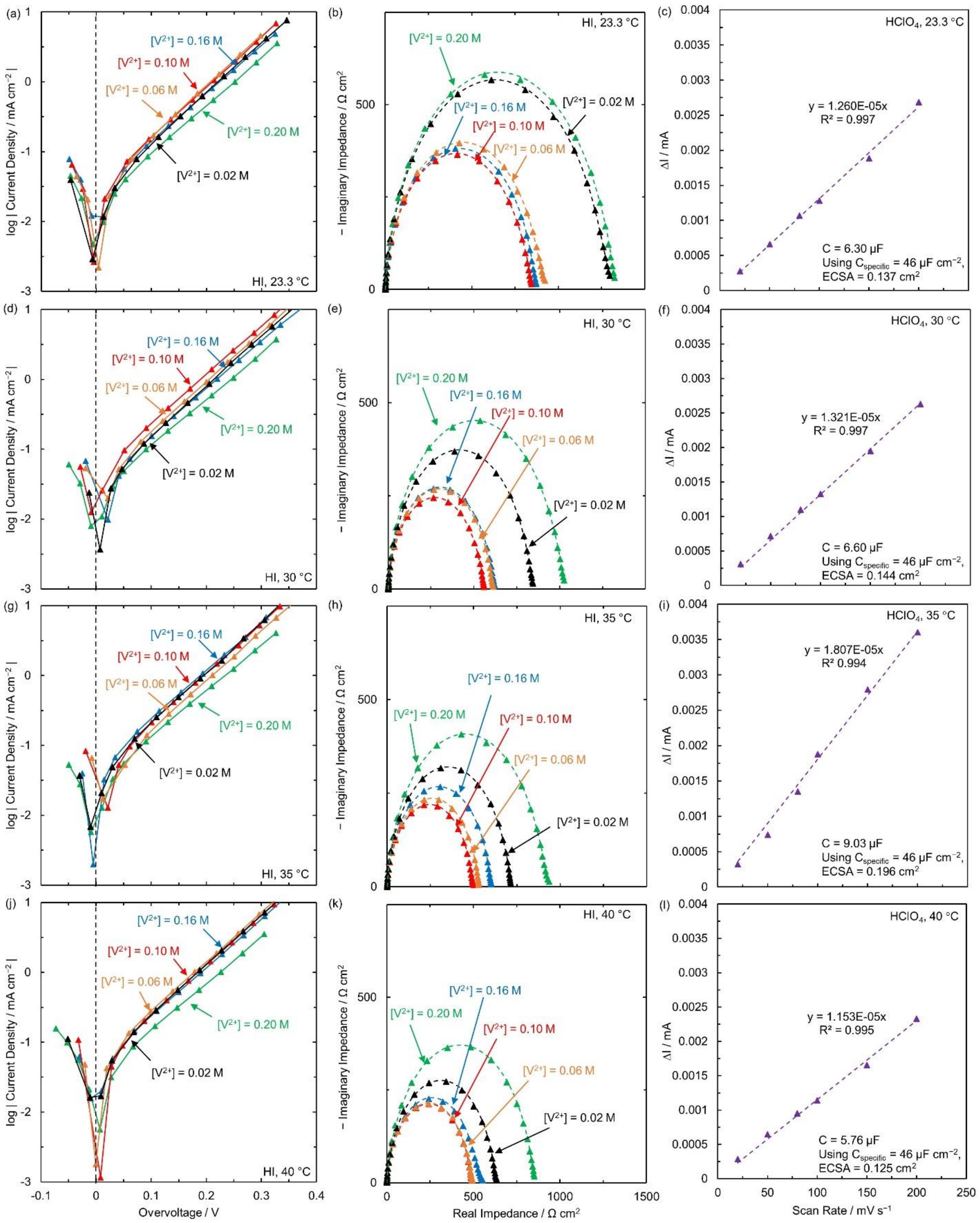

Figure S4. Tafel plots (a, d, g, j) and Nyquist plots (b, e, h, k) at various $[V^{2+}]$ concentrations (0.20, 0.16. 0.10, 0.06, and 0.02 M) at different temperatures (23.3, 30, 35, and 40 °C) in HI on edge plane pyrolytic graphite electrode. Tafel plots are constructed from steady-state measurements corrected for solution resistance. Triangles in Tafel plots correspond to experimental measured points while the lines are used to guide the eye. The triangles in the Nyquist plots are from EIS experimental measurements (b, e, h, k) and the dotted lines represent fits obtained from the EC-

Lab software after modelling as a modified Randles circuit. All fitting parameters are available in Table S4. The ECSA (c, f, i, l) was obtained by the cyclic voltammetry capacitance method in $HClO_4$ for measurements in HI. Note that all these plots are from a single experimental run. Each condition was repeated three times and the average exchange current densities evaluated from the three runs are reported in the main text.

Table S4. Fitted parameters for a single experimental run obtained using the Zfit application in EC-Lab software for HI at various [V^{2+}] concentrations at different temperatures on edge plane pyrolytic graphite electrode. A modified Randles circuit is used for fitting all the EIS measurements. The obtained fits are shown by dotted lines in Nyquist plots and the ECSA is evaluated from cyclic voltammetry capacitance method shown in Figure S4. Each condition was repeated three times and the average exchange current densities evaluated from the three runs are reported.

HI									
T (°C)	[V^{2+}] (M)	R_s (Ω)	Q_{CPE} (F $s^{(a-1)}$)	a	R_{d2} (Ω)	t_{d2} (s)	R_3 (Ω)	ECSA (cm^2)	R_{ct} (Ω cm^2)
23.3	0.20	10.2	3.48×10^{-6}	0.938	199	0.477	9420	0.137	1290.5
	0.16	10.2	3.65×10^{-6}	0.924	365	0.388	6270		859.0
	0.10	10.7	3.36×10^{-6}	0.934	203	0.320	5920		811.0
	0.06	10.7	3.37×10^{-6}	0.928	241	1.085	6480		887.8
	0.02	10.8	3.34×10^{-6}	0.931	254	1.116	9190		1259.0
30.0	0.20	7.08	3.86×10^{-6}	0.937	186	0.519	6930	0.144	997.9
	0.16	7.18	3.87×10^{-6}	0.936	108	0.425	4200		604.8
	0.10	6.83	3.88×10^{-6}	0.935	95	0.293	3780		544.3
	0.06	8.54	3.83×10^{-6}	0.936	101	0.354	4140		596.2
	0.02	12.2	3.77×10^{-6}	0.936	123	0.663	5740		826.6
35.0	0.20	5.17	4.58×10^{-6}	0.940	156	0.531	4560	0.196	893.8
	0.16	4.91	4.58×10^{-6}	0.933	164	0.397	3030		593.9
	0.10	4.92	4.70×10^{-6}	0.932	60	0.458	2500		490.0
	0.06	4.76	4.36×10^{-6}	0.943	60	0.189	2630		515.5
	0.02	4.68	4.31×10^{-6}	0.945	74	0.287	3560		697.8
40.0	0.20	4.86	3.93×10^{-6}	0.919	149	0.566	6740	0.125	842.5
	0.16	4.80	3.90×10^{-6}	0.921	218	0.573	4130		516.3
	0.10	4.73	3.98×10^{-6}	0.918	73	0.340	3870		483.8
	0.06	4.55	3.79×10^{-6}	0.928	86	0.361	3840		480.0
	0.02	4.28	3.88×10^{-6}	0.922	101	0.164	4970		621.3

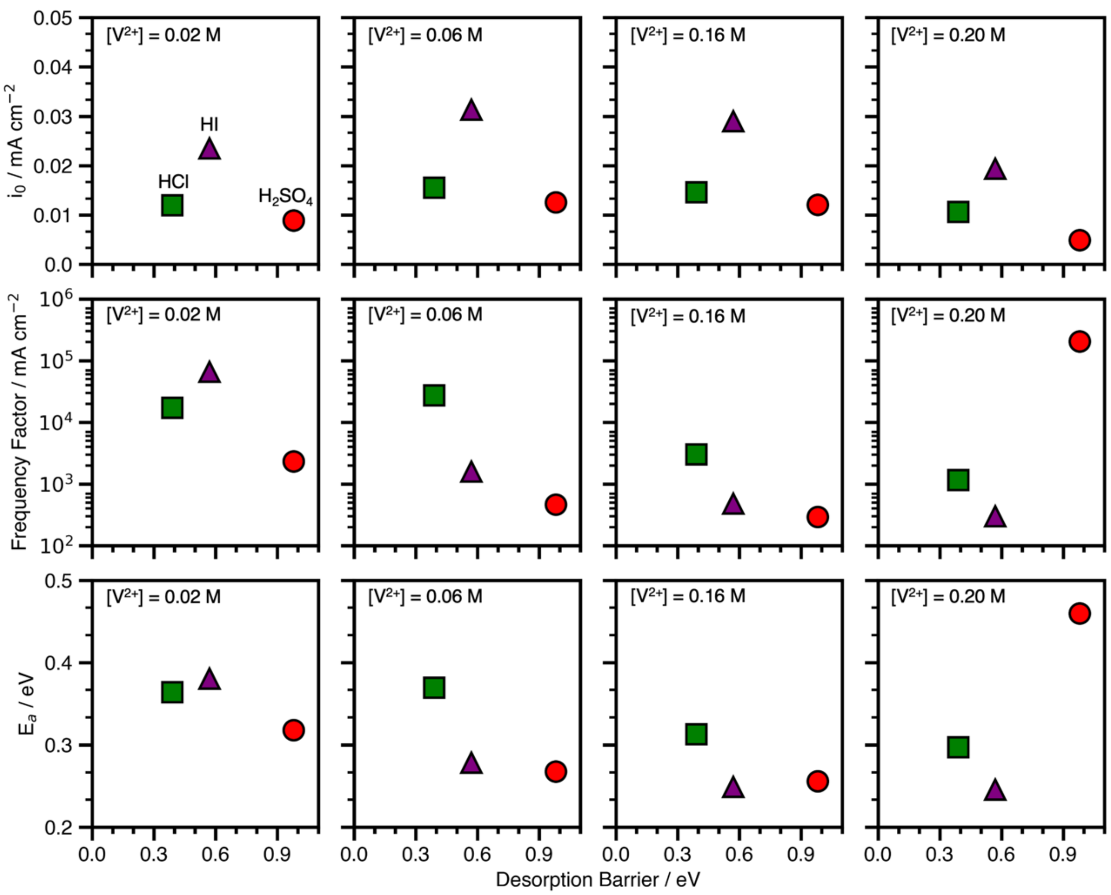

Figure S5. Experimentally evaluated exchange current densities (i_o) at 23.3 °C, apparent frequency factors, and apparent activation energies (E_a) for V^{2+}/V^{3+} on EPPG plotted against the predicted desorption barriers. Experimental parameters were evaluated using the Tafel method at 0.02, 0.06, 0.16, and 0.2 M V^{2+} concentration, with corresponding concentrations of V^{3+} to make the total V ion concentration 0.2 M. Apparent frequency factors and apparent activation energies are from temperature measurements at 23.3, 30, 35, and 40 °C.

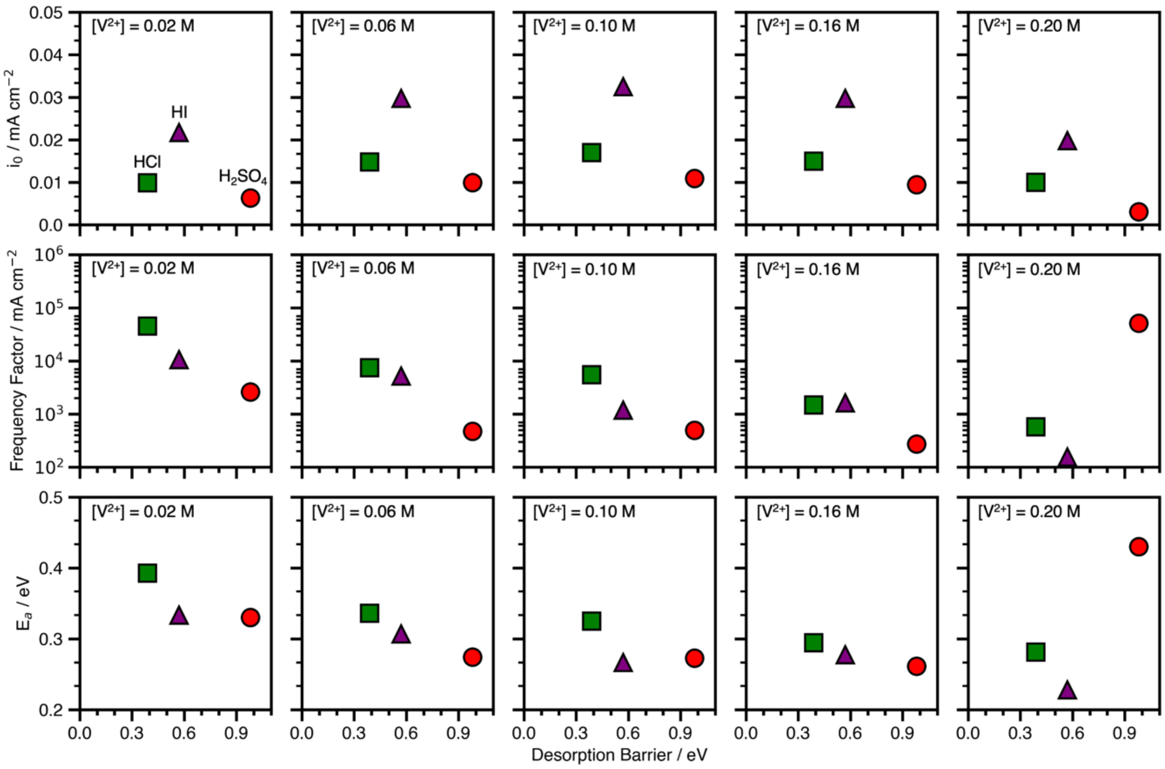

Figure S6. Experimentally measured exchange current densities (i_o) at 23.3 °C, apparent frequency factors, and apparent activation energies (E_a) for V^{2+}/V^{3+} on EPPG as a function of the predicted desorption barrier. Experimental parameters were evaluated using the Charge Transfer Resistance method at 0.02, 0.06, 0.10, 0.16, and 0.2 M V^{2+} concentration, with corresponding concentration of V^{3+} to make the total V ion concentration 0.2 M. Apparent frequency factors and apparent activation energies are from temperature measurements at 23.3, 30, 35, and 40 °C.

S4. Cleaning of EPPG and measurement of ECSA

The EPPG disk insert was first rinsed with Millipore water, followed by polishing for ~ 5 min with ultrafine alumina slurry (0.05 μm diameter particles, Allied Pure) on a Rayon microcloth polishing cloth (Pine Research). Subsequently, the electrode was rinsed with Millipore water multiple times to get rid of any alumina particles suspended on surface, followed by sonication for 45 min. Following sonication, the disk insert was mounted into a Pine Research E5-series ChangeDisk RDE assembly and affixed to a Modulated Speed Rotator.

The cyclic voltammetry capacitance method was used to evaluate the ECSA of the EPPG. Cyclic voltammograms, 10 cycles each, at different scan rates (10, 20, 50, 80, 100, 150, and 200 mV/s) were conducted between −0.1 to 0.4 V (vs. Ag/AgCl) in 1 M H_2SO_4. The difference in the current at 0.15 V (vs. Ag/AgCl) during the increasing and decreasing voltage sweeps from the 10th cycle was plotted against the scan rate. The slope of the corresponding line passing through the origin is twice the capacitance corresponding to the electrochemical double layer. Using a specific capacitance of 40 μF cm^{-2} for carbon surfaces in H_2SO_4,[37,38] the ECSA was determined.

For measurements in HCl and HI, we measured the ECSA in 1 M $HClO_4$ instead of 1 M HCl due to the possibility of specific adsorption of Cl^- and I^- anions, respectively, on electrode surface during cycling. Additionally, H_2SO_4 was not used to prevent the introduction of any SO_4^{2-} due to their inhibiting effect on reaction kinetics.[23] To consider the change in specific capacitance with change in electrolyte, the ECSA of EPPG was measured in 1 M H_2SO_4, followed by 1 M $HClO_4$, and then again in 1 M H_2SO_4, assuming the same specific capacitance. We observed that the ECSAs in 1 M H_2SO_4 initially and after measurements in 1 M $HClO_4$ were within ±2%, indicating that the EPPG surface is unchanged after using

it for measurements in 1 M $HClO_4$. Further, on comparing the ECSA in 1 M H_2SO_4 to 1 M $HClO_4$ using the same specific capacitance, ECSAs were observed to be ~15% higher in 1 M $HClO_4$ compared to 1 M H_2SO_4. As a result, to compensate for this effect, the specific capacitance of 46 μF cm^{-2} (~15% higher than in 1 M H_2SO_4) was used to evaluate the ECSA in 1 M $HClO_4$.

S5. Computational Methods and Simulation Cell Setup for Metadynamics

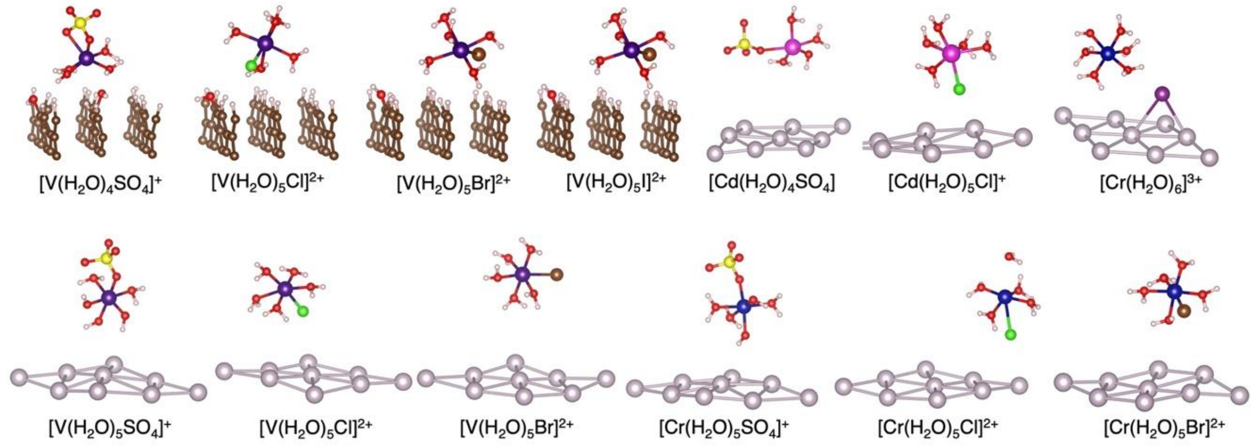

Figure S7. Snapshots of desorbed complexes from metadynamics simulations for graphite($11\bar{2}0$) and Hg(111). Only the top surface layer is shown for clarity. Atom color legend: gray = Hg, brown = C, dark purple = V, pink = Cd, blue = Cr, green = Cl, brown = Br, purple = I, red = O, yellow = S, white = H.

Spin-polarized density functional theory (DFT) calculations were performed using Vienna Ab Initio Simulation Package (VASP).[39–41] The PBE functional was used for all metadynamics simulations.[42] The projector augmented wave method was chosen to describe electron-ion interactions.[43] A 400 eV plane wave kinetic energy cutoff was set for all systems. A Γ-centered 1×1×1 *k*-point grid was used. The systems modeled were V^{3+}-anion complexes on the graphite edge plane ($11\bar{2}0$), V^{3+}-, Cr^{3+}-, and Cd^{2+}-anion complexes on Hg(111), and Fe^{3+}-anion complexes on Au(111).

Details of the unit cell dimensions and compositions for NVT metadynamics simulations are shown in **Figure 2a** and **Figures S8–S9**. Graphite($11\bar{2}0$) with lattice constants of a = 2.45 Å and c = 6.77 Å (**Figure 2a**) was used as a model surface for EPPG. The graphite surface was passivated with 42 adsorbed *H and five adsorbed *OH. In addition to vanadium, 38 explicit water molecules were added to maintain an aqueous environment with a density of 1 g/cm^3. The middle layer of carbon atoms was fixed to their bulk positions. Spin-polarized DFT was used with a spin density of 2 spin-up electrons to model the V^{3+} ion. A three-layer 3×3 Hg(111) surface with an Hg interatomic distance of 3.60 Å (**Figure S8**) was used to model a mercury drop electrode. In addition to the Cr^{3+} ion, 48 explicit water molecules were added to maintain an aqueous environment with a density of 1 g/cm^3. The middle layer of Hg atoms was fixed to their bulk positions. Spin-polarized DFT was used with a spin density of 3 spin-up electrons to model the Cr^{3+} ion. The same procedure was used for the V^{3+} and Cd^{2+} on Hg(111) with 2 spin-up electrons for V^{3+} and 0 spin-up electrons for Cd^{2+}. VASP input files and geometries for metadynamics simulations are available in the NOMAD repository at https://dx.doi.org/ 10.17172/NOMAD/2021.07.03-1

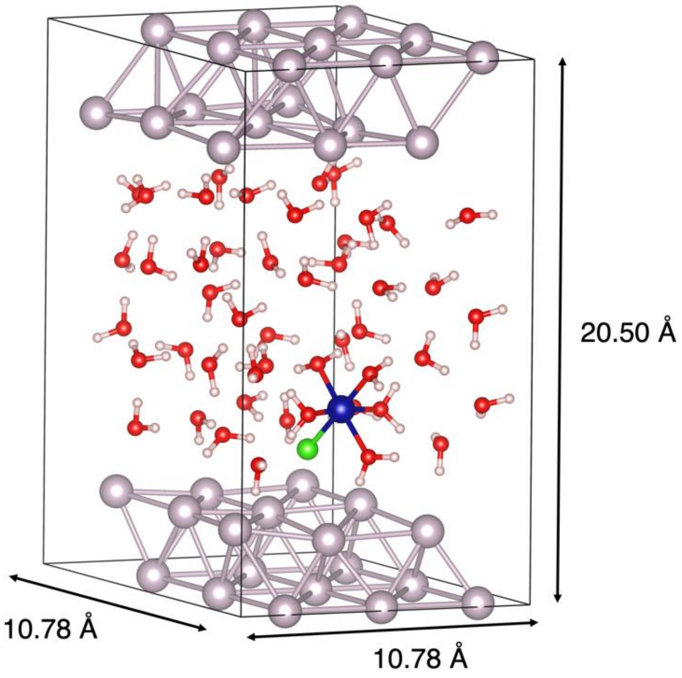

Figure S8. Simulation cell for $[Cr(H_2O)_5X]^{2+}$ on Hg(111). Atom color legend: red = O, gray = Hg, green = anion, beige = H, blue = Cr.

A three-layer 4×4 Au(111) surface with lattice constant a = 4.17 Å (**Figure S9**) was used as a model for a polycrystalline gold electrode. In addition to the $[Fe(H_2O)_5X]^{2+}$ complex, 48 explicit water molecules were added to the simulation cell to maintain an aqueous environment with a density of 1 g/cm^3. To prevent system translation and maintain a symmetric simulation cell, the middle layer of Au atoms was fixed to their bulk positions during the simulation. Spin-polarized DFT was used with a spin density of five spin-up electrons to model the high-spin Fe^{3+} ion.

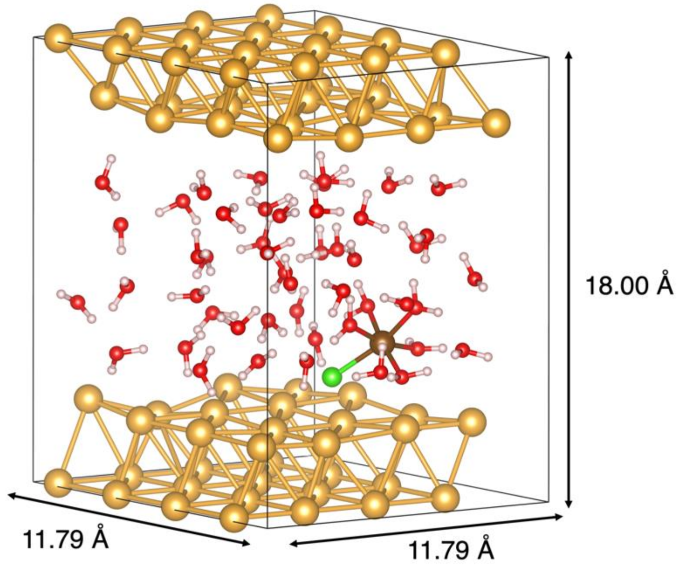

Figure S9. Simulation cell for $[Fe(H_2O)_5X]^{2+}$ complex on Au(111). Atom color legend: red = O, gold = Au, green = anion, beige = H, brown = Fe.

A Nosé-Hoover thermostat was used to equilibrate the system at 300 K.[44,45] Equations of motion were integrated with a time step of 0.5 fs, and all hydrogen atoms were replaced with deuterium to dampen the high frequency OH bond vibrations. To calculate desorption free energy barriers, the distance between the metal ion and the surface (*z* coordinate) was chosen as the collective variable. Adding an additional collective variable that biases the coordination number between the metal ion and solvent water molecules, which occurs on a longer timescale than desorption, did not affect the desorption barriers and thus was not further considered (**Figure S10**).

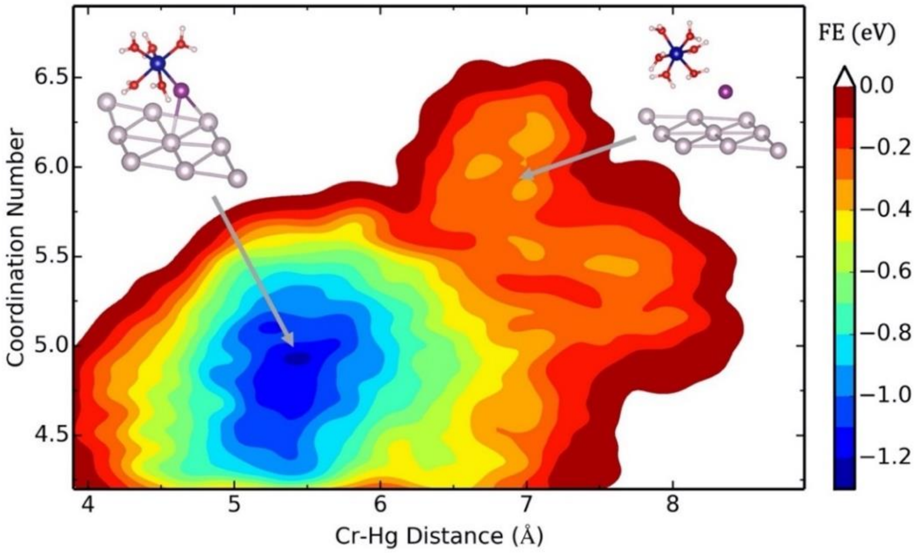

Figure S10. Two-dimensional contour plot showing the free energy (FE) surface of the desorption of $[*I\text{-}Cr(H_2O)_5]^{2+}$ when biasing both coordination number of water and Cr^{3+}-Hg distance. Snapshots of the adsorbed and desorbed complexes with solvent water molecules removed are shown, and the arrows show their position on the free energy surface. The reference state is the desorbed state. Atom color legend: gray = Hg, blue = Cr, purple = I, red = O, white = H.

The free energy barrier for $[*I\text{-}Cr(H_2O)_5]^{2+}$ desorption on Hg(111) when biasing both the coordination number of Cr^{3+} with water and the Cr^{3+} distance from the surface is 1.24 eV (**Figure S10**). The most stable adsorbed $[*I\text{-}Cr(H_2O)_5]^{2+}$ configuration occurs at a Cr^{3+}-Hg distance of 5.2 Å and a coordination number of five and is described by the dark blue energy well. This prediction is similar to the desorption barrier when only biasing the Cr^{3+} distance from the surface, which is 1.21 eV as shown in the free energy profile in **Figure 3b**. The second collective variable biasing the coordination number of water with the metal ion did not change the desorption barriers by more than three percent.

After at least a 5 ps equilibration period, gaussian bias potentials with height 0.01 eV and width 0.10 Å were deposited every 15 fs to bias the collective variable. The width of the gaussian hills was based on the standard deviation of changes in the collective variable during equilibration. Metadynamics simulations were stopped once the distance of the desorbed metal ion complex exceeded $1.5R_{S-X} + R_{X-M}$ from the surface for more than 0.3 ps, where R_{S-X} is the surface-anion bond distance in the gas phase and R_{X-M} is the bond distance between the anion-metal ion of the gas-phase complex (**Table S5**). The effect of applied potential was not considered in these simulations.

Table S5. Bond distances used as desorption barrier cutoffs in metadynamics simulations.

Surface (*S*)	Anion (*X*)	Metal (*M*)	R_{S-X} (Å)	R_{X-M} (Å)
Hg(111)	OH^-	V^{3+}	1.40	1.78
Hg(111)	Cl^-	V^{3+}	2.02	2.17
Hg(111)	Br^-	V^{3+}	2.22	2.32
Hg(111)	I^-	V^{3+}	2.48	2.55
Hg(111)	OH^-	Cr^{3+}	1.40	1.81
Hg(111)	Cl^-	Cr^{3+}	2.42	2.17
Hg(111)	Br^-	Cr^{3+}	2.60	2.33
Hg(111)	I^-	Cr^{3+}	2.79	2.55
Hg(111)	OH^-	Cd^{2+}	1.40	2.07
Hg(111)	Cl^-	Cd^{2+}	2.42	2.38
Au(111)	OH^-	Fe^{3+}	1.31	1.77
Au(111)	Cl^-	Fe^{3+}	1.93	2.15
Au(111)	Br^-	Fe^{3+}	2.08	2.29
graphite($11\bar{2}0$)	OH^-	V^{3+}	1.37	1.78
graphite($11\bar{2}0$)	Cl^-	V^{3+}	1.73	2.17
graphite($11\bar{2}0$)	Br^-	V^{3+}	1.91	2.32
graphite($11\bar{2}0$)	I^-	V^{3+}	2.12	2.55

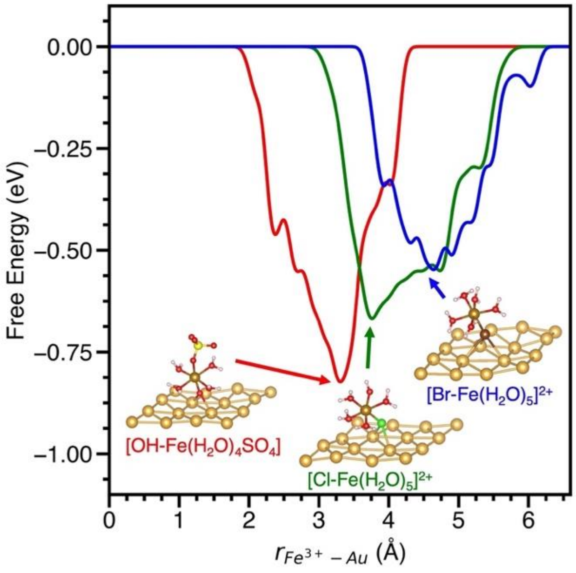

Figure S11. Desorption barriers of Fe^{3+}-anion complexes bound to Au(111) through *OH, *Cl and *Br bridges. Geometries of the adsorbed Fe^{3+}-anion complexes are shown at the lowest point on the free energy profile. Atom color legend: Au = gold, Fe = brown, O = red, S = yellow, H = white, Cl = green, Br = dark brown.

S6. V^{3+} Desorption Barriers Across Different Surfaces

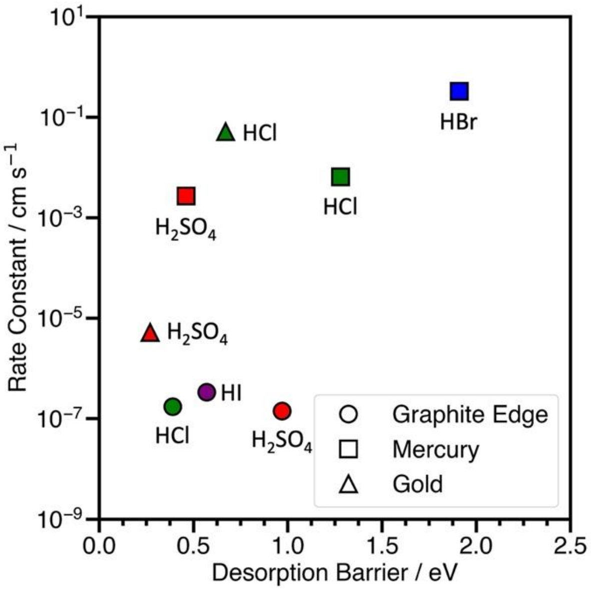

Figure S12. Standard experimental rate constants on EPPG, mercury drop electrodes, and gold vs. the predicted desorption barrier of V^{3+}-anion complex on graphite(11$\bar{2}$0), Hg(111), and Au(111). Colors denote the sulfuric and hydrohalic acid, namely H_2SO_4 (red), HCl (green), HBr (blue), and HI (purple).

References

1 W. Mehl and J. O. M. Bockris, *J. Chem. Phys.*, 1957, **27**, 818–819.

2 A. A. Moussa and H. M. Sammour, *J. Chem. Soc.*, 1960, 2151–2157.

3 V. V. Gorodetskii and V. . Losev, *Elektrokhimiya*, 1966, **2**, 656.

4 G. Salié, *Zeitschrift Fur Phys. Chemie*, 1970, **244**, 1.

5 D. J. Kooijman and J. H. Sluyters, *Electrochim. Acta*, 1967, **12**, 1579–1592.

6 H. P. Agarwal, *Electrochim. Acta*, 1971, **16**, 1395–1414.

7 J. K. Frischmann and A. Timnick, *Anal. Chem.*, 1967, **39**, 507–511.

8 S. Ferro and A. De Battisti, *Electrochim. Acta*, 2002, **47**, 1641–1649.

9 Z. Galus and R. N. Adams, *J. Phys. Chem. A*, 1962, **43**, 5–10.

10 A. B. Sheinin, V. A. Zinovev and V. L. Kheifets, *Russ. J. Phys. Chem. A*, 1961, **35**, 513–516.

11 J. Jones Ulrich and F. C. Anson, *Inorg. Chem.*, 1969, **8**, 195–200.

12 F. C. Anson, N. Rathjen and R. D. Frisbee, *J. Electrochem. Soc.*, 1970, **117**, 477.

13 T. Hurlen, *Acta Chem. Scand.*, 1961, **15**, 621–629.

14 K. Niki and H. Mizota, *J. Electroanal. Chem.*, 1976, **72**, 307–317.

15 L. A. Curtiss, J. W. Halley, J. Hautman, N. C. Hung, Z. Nagy, Y. -J. Rhee and R. M. Yonco, *J.*

Electrochem. Soc., 1991, **138**, 2032–2040.

16 D. H. Angell and T. Dickinson, *J. Electroanal. Chem.*, 1972, **35**, 55–72.

17 J. Weber, Z. Samec and V. Mareček, *J. Electroanal. Chem.*, 1978, **89**, 271–288.

18 M. Stulikova and F. Vydra, 1972, **38**, 349–357.

19 D. Galizzioli and S. Trasatti, *J. Electroanal. Chem.*, 1973, **44**, 367–388.

20 M. Gross and J. Jordan, *Pure Appl. Chem.*, 1985, **57**, 633–638.

21 K. G. Everett, L. A. Drew, S. J. Ericson and G. M. Schmid, *J. Electrochem. Soc.*, 1978, **125**, 389–394.

22 J. O'M Bockris, R. J. Mannan and A. Damjanovic, *J. Chem. Phys.*, 1968, **48**, 1898–1904.

23 V. V. Losev and A. I. Molodov, *Dokl. Akad. Nauk SSSR*, 1963, **148**, 1114–1117.

24 J. N. Gaur and D. S. Jain, *Electrochim. Acta*, 1966, **11**, 1661–1665.

25 A. A. Moussa, M. M. Abou Romia and F. El-Taib Heakal, *Electrochim. Acta*, 1970, **15**, 1391–1397.

26 M. Hollnahel and B. Landsberg, *Zeitschrift Fur Phys. Chemie*, 1959, **212**, 94.

27 J. E. B. Randles, *Progress in Polarography 1*, Interscience, New York, 1962.

28 V. I. Kravtsov, *Russ. Chem. Rev.*, 1976, **45**, 284–297.

29 A. T. Hubbard and F. C. Anson, *The theory and practice of electrochemistry with thin layer cells, in Electroanalytical Chemistry A.J. Bard (Vol. 4), A Series of Advances.*, Marcel Dekker, New York, 1970.

30 H. Agarwal, J. Florian, B. Goldsmith and N. Singh, *Cell Reports Phys. Sci.*, 2021, **2**, 100307.

31 J. Blackledge and N. S. Hush, *J. Electroanal. Chem.*, 1963, **5**, 435–449.

32 J. Koryta, *Electrochim. Acta*, 1962, **6**, 67–74.

33 T. Kambara and T. Ishii, *Rev. Polarogr.*, 1961, **9**, 30–35.

34 H. Agarwal, J. Florian, B. R. Goldsmith and N. Singh, *ACS Energy Lett.*, 2019, **4**, 2368–2377.

35 A. J. Bard and L. R. Faulkner, *Electrochemical Methods: Fundamentals and Applications*, John Wiley & Sons, Inc., 2001.

36 10.38, Ed., *EC-Lab Software User's Manual*, BioLogic Science Instruments, 2014.

37 J. P. Randin and E. Yeager, *J. Electroanal. Chem.*, 1975, **58**, 313–322.

38 P. Iamprasertkun, W. Hirunpinyopas, A. Keerthi, B. Wang, B. Radha, M. A. Bissett and R. A. W. Dryfe, *J. Phys. Chem. Lett.*, 2019, **10**, 617–623.

39 G. Kresse and J. Furthmüller, *Phys. Rev. B*, 1996, **54**, 11169–11186.

40 G. Kresse and J. Furthmüller, *Comput. Mater. Sci.*, 1996, **6**, 15–50.

41 G. Kresse, *J. Non. Cryst. Solids*, 1995, **192–193**, 222–229.

42 J. P. Perdew, K. Burke and M. Ernzerhof, *Phys. Rev. Lett.*, 1996, **77**, 3865–3868.

43 D. Joubert, *Phys. Rev. B - Condens. Matter Mater. Phys.*, 1999, **59**, 1758–1775.

44 W. Hoover, *Phys. Rev. A*, 1985, **31**, 1695–1697.

45 S. Nosé, *J. Chem. Phys.*, 1984, **81**, 511–519.
